# Soft tissue surface deformation detection in endoscopic images

**DOI:** 10.1016/j.isci.2026.117258

**Published:** 2026-08-22

**Authors:** Ranyang Li, Ziyu Zhu, Nan Wei, Wufeng Liu, Xiaodong Guo, Chao Fan, Junjun Pan

**Affiliations:** 1 School of Artificial Intelligence and Big Data, Henan University of Technology, Zhengzhou 450001, China; 2 Henan Provincial People’s Hospital, Zhengzhou University, Zhengzhou 450003, China; 3 State Key Laboratory of Virtual Reality Technology and Systems, Beihang University, Beijing 100191, China

**Keywords:** Deformation description, Deformation detection, Endoscopic surgical navigation

## Abstract

Soft tissue surface deformation in endoscopic images breaks the alignment between preoperative models and the intraoperative view, which affects reconstruction, registration, and feature matching in minimally invasive surgery. We present a unified framework that detects and tracks these deformations. A gradient-density formulation locates deformation regions from inflection points of the gradient information-density distribution, without handcrafted priors or manual labels. A self-attention residual encoder-decoder then jointly produces deformation contours and a compact latent representation, and a deformation-aware tracker fuses contour, semantic, and color cues to maintain temporal consistency. Across in-house endoscopic sequences and public segmentation, boundary, and tracking benchmarks, the method attains higher accuracy and more complete, connected contours than gradient operators and existing segmentation models. These stable deformation cues can support feature matching, registration, and overlay stability in augmented and mixed reality surgical navigation.

## Highlights

• Gradient-density method enables unsupervised soft-tissue deformation detection
• Self-attention residual network jointly segments and encodes latent features
• Deformation-aware tracking fuses contour, semantic, and color cues over time
• High accuracy on in-house and public benchmarks supports AR/MR navigation

## Graphical abstract

**Figure:**
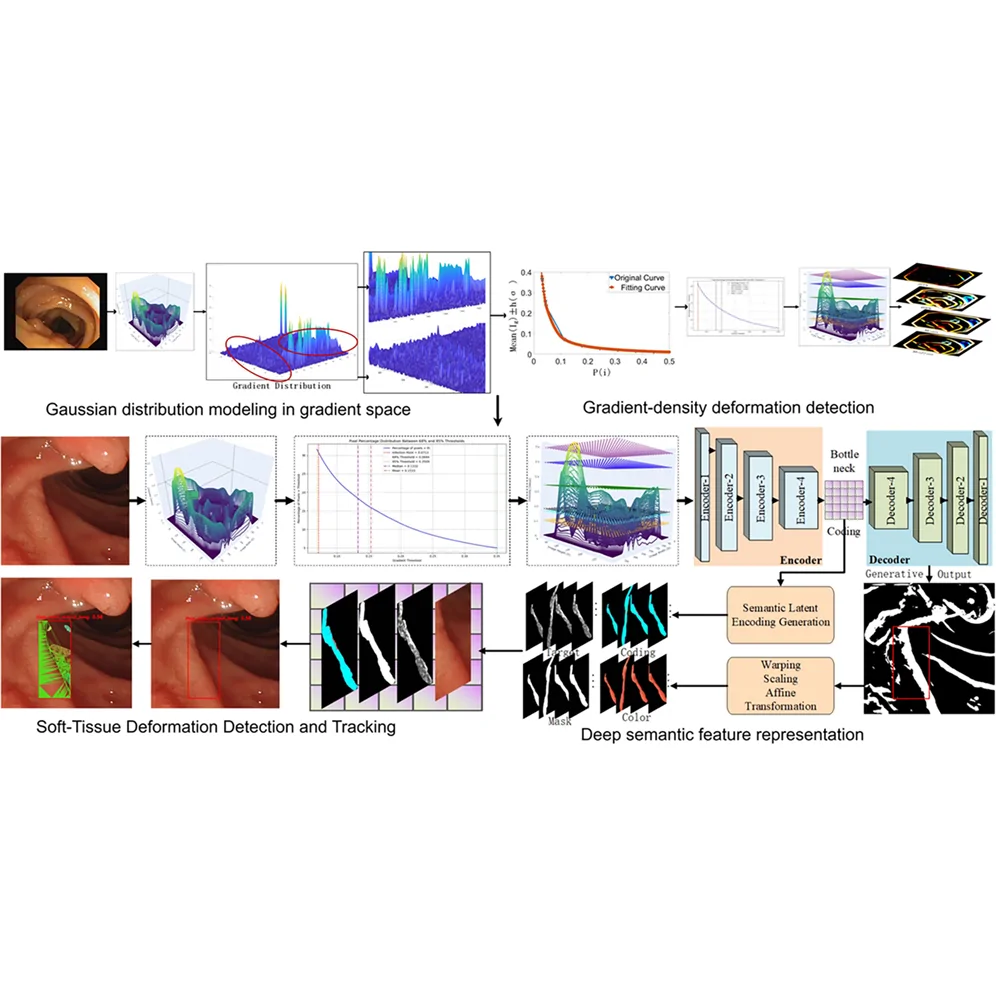

## Teaser

Surgery; Computer application in medicine; Medical imaging; Artificial intelligence; Navigation system

## Introduction

**Figure 1:**
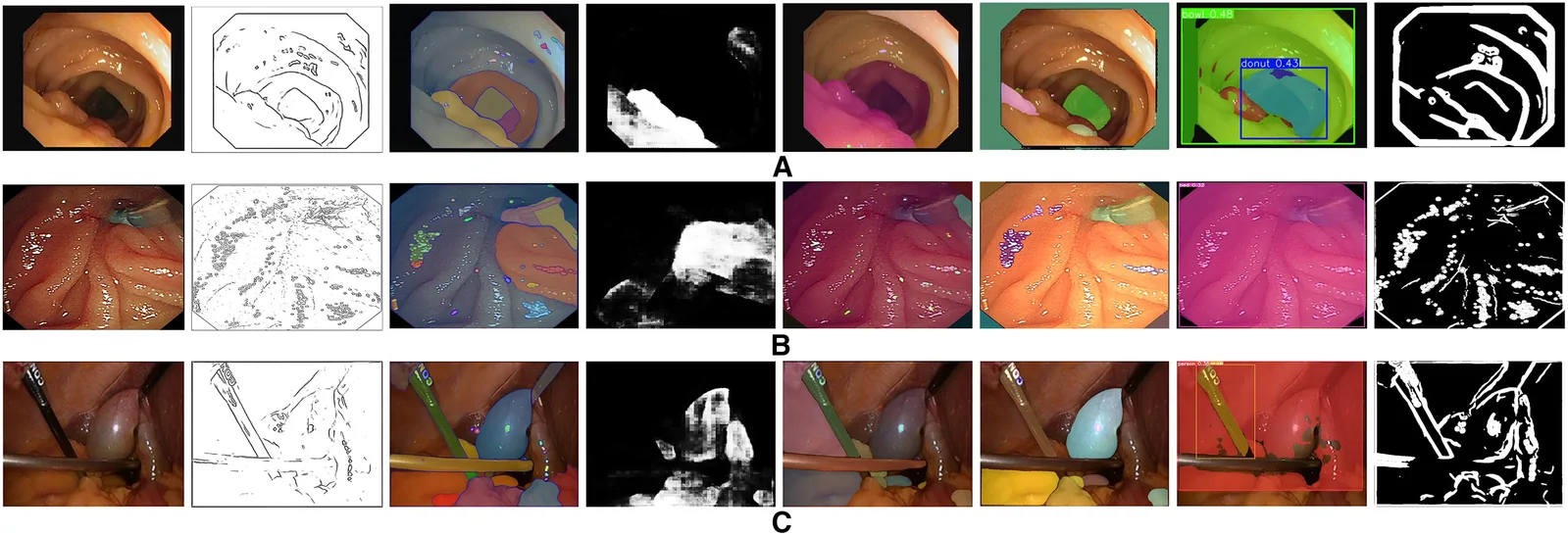
Detection of soft tissue deformations by foundation and learning-based models The leftmost column shows input images from three minimally invasive surgical scenarios. Columns 1–6 display results from TEED, SAM, CaraNet, YOLOV9, TinySAM, and SAM-Track. The rightmost column presents results from the proposed method, showing more consistent delineation of deformation-related contour regions in these challenging cases.

**Figure 2:**
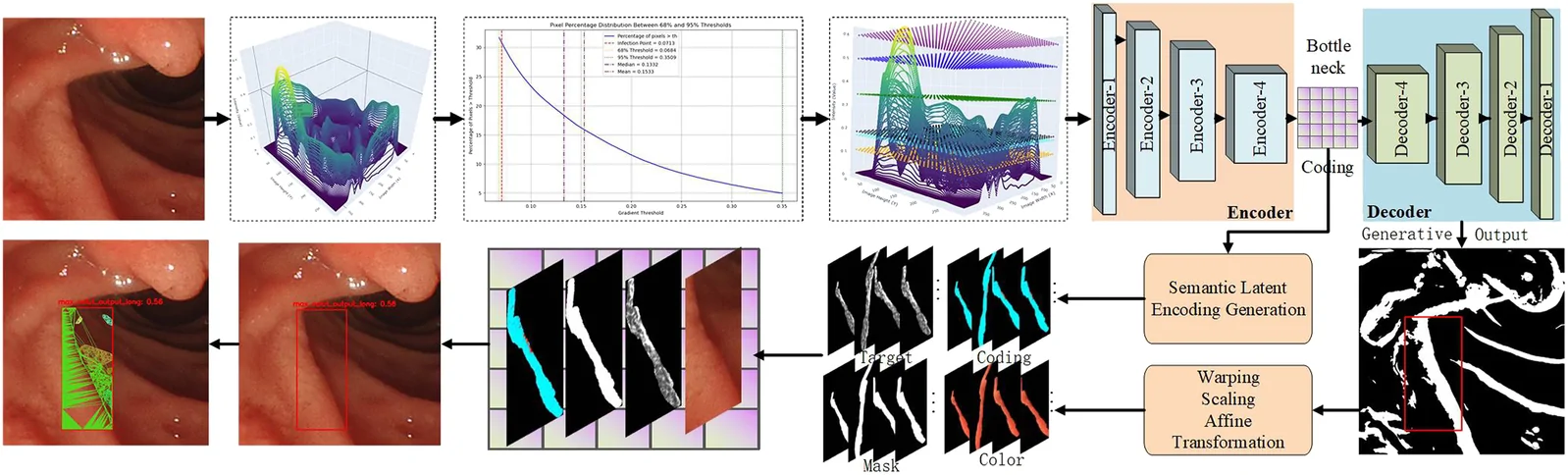
The framework of the proposed method

**Figure 3:**
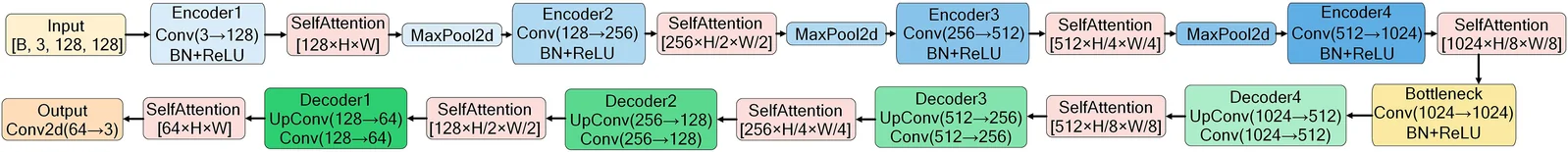
End-to-end multitask framework with self-attention and residual modules for deformation segmentation and latent encoding in endoscopic imaging

**Figure 4:**
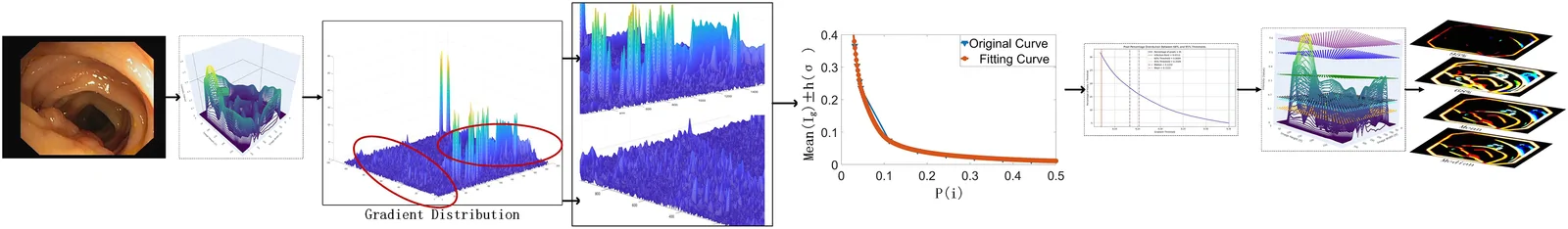
Visualization of gradient information after Gaussian filtering (*x*, *y*) represent the spatial coordinates, *z* represents the intensity at each pixel, and colored planes indicate the division between low- and high-frequency components based on different inflection points.

Accurate detection of soft tissue surface deformations in endoscopic imagery remains a fundamental yet unresolved challenge, particularly in anatomical regions such as the gastrointestinal tract, where continuous peristaltic motion induces frequent, large-magnitude, and highly non-rigid surface variations. These deformations, characterized by dynamic folding, stretching, and compression patterns, profoundly alter the geometric and photometric properties of tissue surfaces, making reliable deformation detection extremely difficult. In the context of augmented and mixed reality (AR/MR)-assisted surgical navigation, failure to accurately detect these deformation regions significantly undermines downstream processes, including deformation tracking, feature descriptor extraction and correspondence matching, as well as non-rigid registration between preoperative models and intraoperative 3D reconstructions. Moreover, deformation-dense regions with pronounced folds often lead to sparse and incomplete geometric reconstructions, thereby reducing spatial precision and overall navigation reliability.^1^^,^^2^ Consequently, robust and precise deformation detection becomes a prerequisite for achieving geometrically consistent perception and reliable scene understanding in AR/MR-assisted endoscopic surgery. In this paper, “deformation detection” refers specifically to identifying soft-tissue regions undergoing nonrigid surface motion (e.g., peristalsis, folding, stretching, traction), whose visual signature is dense and temporally evolving contour/edge patterns. Rather than treating these outputs as generic edge extraction, we use deformation-related contours as structured cues and further encode them into deformation-aware descriptors for downstream tracking, matching, and navigation-related tasks. Recent advances in implicit neural representations and differentiable rendering have significantly improved the efficiency and fidelity of 2D and 3D scene reconstruction through end-to-end optimization. However, most existing methods still depend on strong priors such as precise camera poses, depth maps, or dense surface correspondence information that typically requires computationally intensive preprocessing or specialized hardware acquisition. This dependency severely constrains their scalability and limits their practical deployment in clinical environments. To overcome these limitations, recent studies have explored contour- or boundary-based differentiable rendering approaches that reconstruct deformable surfaces by minimizing discrepancies between rendered and observed image edges, thus alleviating the need for explicit geometric supervision. Nevertheless, the accuracy of such methods remains highly sensitive to the quality and reliability of deformation-related cues within the input imagery. A central open problem, therefore, lies in the robust detection of soft tissue surface deformations, which can provide stable and geometry-aware priors for implicit representation learning and differentiable rendering frameworks. Achieving reliable deformation detection is essential not only for enhancing reconstruction accuracy and temporal consistency, but also for improving the robustness, interpretability, and clinical feasibility of AR/MR-guided surgical navigation systems.Target segmentation and tracking are fundamental tasks in medical image analysis. With the emergence of large-scale vision models, the field has progressed from isolated tasks toward multimodal data integration and comprehensive scene understanding. However, despite their promise, current large models still suffer from limited accuracy, interpretability, and generalization, particularly when applied to highly deformable biological tissues. Constructing large annotated datasets for modeling deformation remains extremely labor-intensive, and even state-of-the-art frameworks such as SAM, SAM-Track, and TinySAM struggle to capture the intricate non-rigid motion and deformation patterns of soft tissues. As illustrated in Figure 1, these models often yield inconsistent or unstable results on endoscopic imagery due to severe illumination variations, occlusions, and complex surface folding. Consequently, accurate detection of subtle and dynamic soft tissue deformations remains an unresolved challenge in medical image understanding. Currently, no existing method can reliably detect and characterize soft tissue surface deformations with both geometric precision and temporal consistency. To address this gap, we propose a general end-to-end deformation detection framework that jointly performs deformation segmentation and tracking through a contour-aware representation (Figure 2). Our method employs a transformer-based encoder-decoder architecture augmented with residual and MLP modules to learn implicit deformation features (Figure 3). By integrating contour-derived structural cues with semantic embeddings and color information, the framework achieves robust, interpretable, and geometry-consistent detection of soft tissue surface deformations across challenging endoscopic scenarios (Figure 4). The key contributions are as follows.

• We propose a data-driven deformation detection method for accurately localizing soft tissue surface deformation regions. The method leverages inflection points in the information density distribution function derived from the image gradient space to adaptively identify deformation regions. This formulation enables data-driven, unsupervised deformation detection without reliance on handcrafted priors or auxiliary geometric inputs.
• We propose a hybrid transformer-based encoder-decoder architecture that integrates transformer blocks, residual connections, and MLP modules to jointly capture global and local representations of soft tissue deformations. This design enables compact yet discriminative encoding of deformation features, facilitating accurate and efficient matching of deformation regions. The network is trained end-to-end solely on deformation data, without relying on manual annotations or external supervision, ensuring both scalability and robustness in capturing complex non-rigid tissue motions.
• We develop a deformation-aware detection framework that integrates contour-based structural cues, semantic latent embeddings, and color features to achieve robust and geometry-consistent monitoring of soft tissue surface deformations. By constructing a multi-level semantic representation and explicitly modeling the spatiotemporal context of deformations, the framework enables precise detection, feature description, correspondence matching, and temporally coherent tracking. This approach ensures high tracking accuracy even under complex non-rigid tissue motions, illumination variations, and anatomical variability, while maintaining close alignment with the deformation detection outputs.

Our research relates to prior work on deformation feature description and deformation target detection/tracking in medical image analysis. We briefly review previous works in the following categories.

### Deformation feature descriptors

Deformation feature descriptors are fundamental in medical image analysis, particularly for capturing soft tissue deformations in endoscopic imagery. Accurate tracking and characterization of such deformations depend on the extraction of stable, discriminative, and geometry-aware features. Traditional approaches include handcrafted descriptors such as SIFT, SURF, and ORB, as well as gradient-based operators including Roberts, Sobel, Laplacian, Canny, and DoG, which are effective for edge and contour detection. However, these methods are highly sensitive to noise, specular reflections, and geometric deformations, and often rely on heuristic thresholds that limit robustness in complex surgical environments. Recent studies have explored dense and geometric feature descriptors to improve robustness. For instance, Lu et al.^3^ introduced geometric encoding for dense feature construction, Wang et al.^4^ leveraged regional cues for AR-assisted navigation, Asaturyan et al.^5^ combined morphological and multi-level geometric features for pancreas segmentation, and Li et al.^2^ proposed a contour-based descriptor utilizing multi-scale dense features in Euclidean space. While effective in controlled scenarios, classical descriptors struggle under highly deformable and folded tissue surfaces and are difficult to integrate into deep learning frameworks due to their non-differentiable nature. Learning-based approaches, particularly those leveraging large-scale foundation models, have shown promising performance in medical image analysis. Early methods such as fully convolutional networks (FCNs)^6^ and U-Net^7^ laid the foundation for semantic segmentation. Subsequent efforts, including RCF,^8^ Bi-Directional Cascade Networks,^9^ and semantic boundary learning,^10^ improved edge detection precision. More recent models integrate advanced strategies: Liu et al.^11^ introduced graph-based matching, Wang et al.^12^ developed Endo-FM, a foundation model trained on large-scale endoscopic video datasets, and interactive frameworks such as Segment Anything^13^ and SAMTrack^14^ enable segmentation and tracking, while efficient architectures such as CFPNet^15^ and TinyEdge^16^ enhance computational feasibility. In our experiments, we evaluate representative gradient-based operators (Sobel, Laplacian, Canny, and DoG) as classical baselines for contour detection. For learning-based methods, we include Med-SAM,^17^^,^^18^ a medical domain adaptation of SAM; YOLOv11^19^ (yolo11s-seg and yolo11m-seg variants), a one-stage segmentation framework; FastSAM and SAM2, which extend foundation models for efficient and video-based segmentation; TEED,^16^ an efficient edge detection network; CFPNet-M^15^, a lightweight contour detection architecture; CaraNet,^20^ which combines contextual attention for road extraction; and BDCN,^9^ a bi-directional cascade network for edge detection. These baselines represent diverse methodological categories, enabling comprehensive evaluation across traditional and modern approaches. Despite these advances, learning-based models still face significant challenges: constructing annotated datasets for soft tissue surfaces is labor-intensive due to ambiguous structures and weak texture, increasing network depth can lead to over-parameterization and limited interpretability, and current models lack effective mechanisms for capturing fine-grained contours and subtle deformations in highly non-rigid and folded tissue regions. Consequently, designing deformation-specific feature descriptors remains critical for robust soft tissue analysis and AR/MR-assisted surgical navigation.

### Deformation target detection/tracking

Deformation target detection and tracking is a fundamental yet challenging problem in minimally invasive surgery (MIS), where soft tissue surfaces exhibit large non-rigid deformations, low texture, and ambiguous boundaries. Existing methods span from classical correlation filters to modern learning-based approaches, but most struggle to handle these domain-specific challenges. Early techniques, such as minimum output sum of squared error (MOSSE), kernel correlation filter (KCF), spatially regularized DCF (SRDCF), and DSST,^21^^,^^22^ provide computational efficiency but are limited under substantial appearance variations. Xu et al.^23^ enhanced robustness via joint feature selection across spatial and channel domains, partially alleviating sensitivity to deformation. Recent learning-based approaches have further advanced performance through deep architectures. Mask R-CNN^24^ and its derivatives^25^ enable accurate instance segmentation, while point cloud-based methods^26^ and temporal keypoint refinement^27^ improve tracking of dynamically deforming targets. Liu et al.^28^ introduced the TAO-OW benchmark for open-world tracking, emphasizing the need for models that generalize to unseen objects and deformations. Despite these improvements, conventional and learning-based trackers often fail in endoscopic scenarios due to soft tissue non-rigidity, low contrast, and rapid, complex deformations. To address these limitations, task-specific solutions have been proposed. Lu et al.^3^ and Wang et al.^4^ leverage geometric cues and dense regional features to construct more stable representations. Penza et al.^29^ incorporate optical flow for long-term safety region tracking, while endoscopy-specific techniques target polyps,^30^ bleeding,^31^ instruments,^32^ and needle tips.^33^ Luo et al.^34^ proposed a monocular 6-DoF tracking framework based on stochastic filtering, and Cartucho et al.^35^ introduced the SurgT benchmark dataset for evaluating soft-tissue tracking under surgical conditions. In our comparative evaluation, we assess representative tracking and detection baselines across multiple methodological categories: KCF,^36^ a kernelized correlation filter tracker; CREST,^37^ which integrates spatial regularization into correlation filters; AdNET,^38^ an action-driven network for visual tracking; Mask R-CNN,^24^ a two-stage instance segmentation framework; and DDP,^39^ a dense deformation-based propagation method. For tracking-specific evaluation, we additionally compare against CSRT and MOSSE (correlation filter-based trackers), SAM^13^ (foundation model), YOLO-Seg (one-stage segmentation), and transformer-based architectures including OSTrack,^40^ MixFormer,^41^ LMTrack,^42^ and Cutie.^43^ These baselines span traditional correlation filters, foundation models, one-stage detectors, and modern transformer-based trackers, providing comprehensive coverage of the tracking landscape. Despite these advances, robust detection and tracking of deformable, low-texture soft tissue targets in MIS remains an open and critical research problem, motivating the development of deformation-aware target representations and spatiotemporal tracking frameworks capable of handling complex non-rigid motions.

## Results

### Implementation and compute

All experiments were implemented in Python, with OpenCV used for image processing and evaluation, and executed on a virtualized cloud platform equipped with an NVIDIA A100 GPU.

### Datasets and tasks

**Figure 5:**
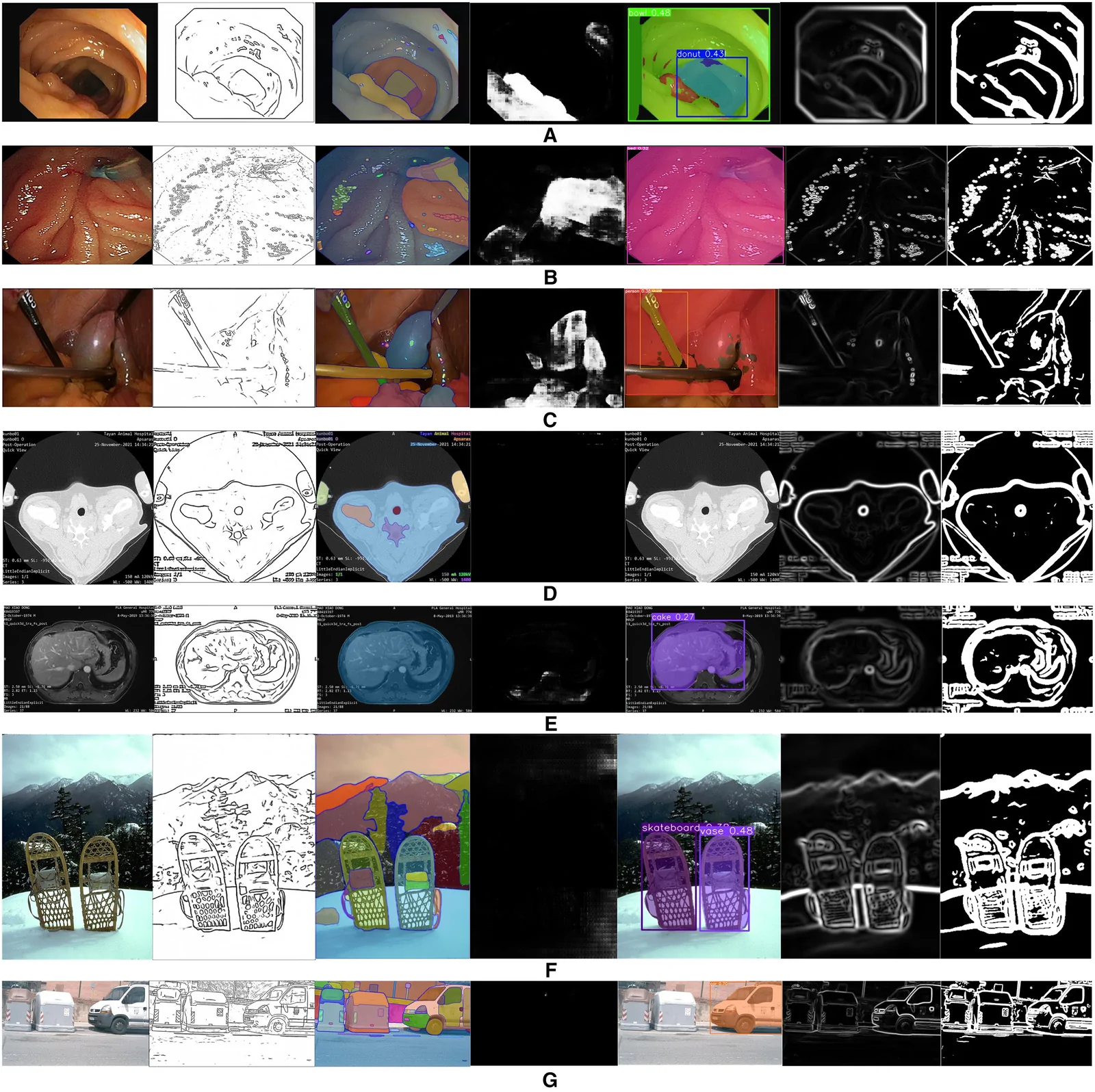
Generalization and universality of the proposed method The first column shows image sequences from different scenarios, including endoscopic surgery scenes, CT/MR medical images, and natural scene images. Columns 2–5 present the segmentation results of TEED,^16^ SAM,^13^ CaraNet,^20^ and YOLOv11,^19^ respectively. Columns 6 and 7 correspond to the results of our method using first-order and second-order contours, respectively.

Our evaluation spans both surgical and generic imagery and targets three complementary aspects: (1) binary foreground-background mask segmentation, (2) boundary/edge (including semantic boundary) detection, and (3) deformation-aware target detection/tracking in endoscopic videos. Contours are critical visual cues for deformation analysis and contour-guided downstream applications; accordingly, we assess not only pixel-level agreement but also structural completeness and robustness under deformation and appearance variation. For surgical deformation analysis, we use three endoscopic sequences with distinct deformation patterns: duodenal papilla (128 frames), intestine with peristaltic motion (164 frames), and relatively planar mucosal folds (206 frames). These sequences are used for qualitative deformation-aware tracking/detection analysis, pixel-level deformation-region segmentation evaluation (Table 3), and match-based tracking/detection comparison. To probe cross-domain generalization, we additionally include CT/MR medical scans and natural-scene images for qualitative verification (Figure 5). For public quantitative benchmarking, we evaluate binary segmentation on two surgical datasets: CholecSeg8k (archive; 8,080 frames) under two foreground definitions (three-organ union and soft-tissue union) and Kvasir-Instrument (590 images) for instrument segmentation (Table 2). For boundary/edge evaluation, we use BIPEDv2 (200 train/50 test) with binary edge maps (Table 1), BSDS500 (200/100/200 train/val/test) with multi-annotator boundaries (union of annotators; Table 4), and SBD (8,498 train/2,857 val) with semantic category boundaries from GTcls (union over present categories; Table 5).

**Table 1:** Boundary precision/recall/F1 (higher is better) on BIPEDv2 with binary edge maps

	BIPEDv2 train (200)			BIPEDv2 test (50)		
Method	B-Prec *↑*	B-Rec *↑*	B-F1 *↑*	B-Prec *↑*	B-Rec *↑*	B-F1 *↑*
ours_th68	0.615_±.10_	0.310_±.10_	0.402_±.09_	0.647_±.09_	0.322_±.11_	0.421_±.11_
ours_th90	$0.670_{\pm .10}^{**}$	$0.205_{\pm .08}^{**}$	$0.305_{\pm .09}^{**}$	$0.699_{\pm .08}^{**}$	$0.214_{\pm .08}^{**}$	$0.319_{\pm .10}^{**}$
ours_th95	$0.681_{\pm .10}^{**}$	$0.187_{\pm .08}^{**}$	$0.285_{\pm .09}^{**}$	$0.709_{\pm .08}^{**}$	$0.194_{\pm .08}^{**}$	$0.296_{\pm .10}^{**}$
sobel	$0.408_{\pm .12}^{**}$	$0.709_{\pm .11}^{**}$	$0.500_{\pm .11}^{**}$	$0.450_{\pm .12}^{**}$	$0.714_{\pm .09}^{**}$	$0.537_{\pm .10}^{**}$
laplacian	$0.290_{\pm .08}^{**}$	$0.704_{\pm .10}^{**}$	0.400_±.09_	$0.320_{\pm .08}^{**}$	$0.700_{\pm .08}^{**}$	0.430_±.08_
canny	$0.510_{\pm .13}^{**}$	$0.452_{\pm .12}^{**}$	$0.459_{\pm .09}^{**}$	$0.556_{\pm .12}^{**}$	$0.468_{\pm .13}^{**}$	$0.492_{\pm .10}^{**}$
dog	$0.282_{\pm .08}^{**}$	$0.687_{\pm .10}^{**}$	0.389_±.08_	$0.307_{\pm .07}^{**}$	$0.689_{\pm .08}^{**}$	0.417_±.08_
yolo_yolo11s-seg	$0.525_{\pm .32}^{**}$	$0.014_{\pm .02}^{**}$	$0.026_{\pm .03}^{**}$	$0.505_{\pm .29}^{**}$	$0.018_{\pm .02}^{**}$	$0.033_{\pm .03}^{**}$
yolo_yolo11m-seg	$0.527_{\pm .31}^{**}$	$0.015_{\pm .02}^{**}$	$0.028_{\pm .03}^{**}$	$0.519_{\pm .30}^{*}$	$0.019_{\pm .02}^{**}$	$0.035_{\pm .03}^{**}$
fastsam_FastSAM-x	$0.142_{\pm .07}^{**}$	$0.037_{\pm .03}^{**}$	$0.055_{\pm .04}^{**}$	$0.140_{\pm .07}^{**}$	$0.031_{\pm .03}^{**}$	$0.048_{\pm .03}^{**}$
ultra_sam_sam2.1_b	$0.252_{\pm .10}^{**}$	$0.045_{\pm .03}^{**}$	$0.072_{\pm .03}^{**}$	$0.249_{\pm .10}^{**}$	$0.046_{\pm .03}^{**}$	$0.071_{\pm .04}^{**}$
ultra_sam_sam2.1_l	$0.254_{\pm .09}^{**}$	$0.063_{\pm .03}^{**}$	$0.097_{\pm .03}^{**}$	$0.255_{\pm .09}^{**}$	$0.060_{\pm .03}^{**}$	$0.093_{\pm .04}^{**}$

Values are mean_±SD_ over images (*n* = 200 train, *n* = 50 test); bold marks the best mean per column. Superscripts denote the significance of the paired two-sided Wilcoxon signed-rank test versus ours_th68 (∗*p* < 0.05, ∗∗*p* < 0.01; no marker: not significant). All methods are evaluated on binarized predictions. Tolerance is set to *t* = 1 pixel in the evaluation scripts.

### Standard controllable dataset for tracking evaluation

To enable objective and reproducible tracking evaluation under controlled conditions, we construct a standardized chessboard-based dataset comprising 11 video sequences (3,848 frames total) captured under controlled imaging conditions with systematic variations in illumination, occlusion, and camera viewpoint. This dataset provides pixel-accurate ground-truth bounding boxes and binary masks generated automatically through chessboard corner detection with sub-pixel precision, eliminating manual annotation subjectivity. Three chessboard specifications (11 × 8 squares at 11 mm, 9 × 8 squares at 8 mm, and 9 × 6 squares at 5 mm) are employed to assess scale robustness. We evaluate our tracker against established methods including a correlation filter (CSRT), foundation models (SAM), one-stage segmentation (YOLO-Seg), and transformer-based architectures (OSTrack, MixFormer, LMTrack, Cutie). Performance is quantified using coverage-aware BBox IoU, Precision Rate, Success Rate, and Center Error metrics. The complete evaluation protocol and quantitative results are presented in Section 0.1 (Table 6).

### Evaluation protocol and baselines

To ensure task-level comparability across datasets and methods with different label spaces and output formats, we evaluate all methods against unified target representations. For mask segmentation, all ground truth annotations are converted to a single binary mask under a fixed foreground definition, and predictions with multiple instances are merged by union. We report Dice and IoU for region overlap, and Boundary F1 (B-F1) for contour fidelity using a tolerance proportional to image scale, $t=\max\;\left(1,round\left(0.0075\cdot \sqrt{H^{2}+W^{2}}\right)\right)$ pixels. For boundary/edge benchmarks (BIPEDv2/BSDS500/SBD), predictions are binarized and evaluated as 1-pixel boundaries using B-Prec/B-Rec/B-F1 with a fixed matching tolerance of *t* = 1 pixel; dataset-level scores are computed as means over per-image metrics (with method-specific counts when predictions are missing). For in-house deformation-region segmentation, we compute SSIM and pixel-level metrics (accuracy, precision, recall, and F1 score) against manual deformation masks (Table 3); following reviewer suggestions, detection-only baselines are used for qualitative comparison and are not included in this pixel-level table. For deformation-aware target detection/tracking, we compare with representative tracking/detection baselines, including KCF,^36^ CREST,^37^ AdNET,^38^ Mask R-CNN,^24^ and DDP.^39^ For contour/edge detection, we compare against (1) traditional operators (Sobel, Laplacian, Canny, and DoG) and (2) learning-based methods including Med-SAM,^17^^,^^18^ YOLOv11^19^ (yolo11s-seg and yolo11m-seg variants), FastSAM, and SAM2, with quantitative evaluations on multiple boundary benchmarks (Tables 1, 2, 3, 4, and 5). For diverse-scenario verification, we further include representative segmentation frameworks such as TinySAM, TEED,^16^ SAM^13^ (and SAM-Track when applicable), CFPNet-M^15^, CaraNet,^20^ and BDCN^9^; representative results on endoscopy/CT-MR/natural scenes are shown in Figure 5. When segmentation-based baselines are used for edge/boundary evaluation, their outputs are converted from masks to 1-pixel boundaries for cross-task comparison.

**Table 2:** Mean Dice/IoU/Boundary F1 for mask-producing methods across three binary segmentation settings

	CholecSeg8k (archive), three-organ (8080)			CholecSeg8k (archive), soft-tissue (8080)			Kvasir-instrument (590)		
Method	Dice *↑*	IoU *↑*	B-F1 *↑*	Dice *↑*	IoU *↑*	B-F1 *↑*	Dice *↑*	IoU *↑*	B-F1 *↑*
ours_th68_fill	0.425_±.18_	0.287_±.15_	0.141_±.13_	**0.769**_±.13_	**0.641**_±.16_	0.269_±.12_	0.233_±.19_	0.148_±.15_	0.168_±.10_
medsam_vit_b	$0.757_{\pm .09}^{**}$	$0.617_{\pm .11}^{**}$	$0.260_{\pm .09}^{**}$	$0.736_{\pm .06}^{**}$	$0.586_{\pm .08}^{**}$	$0.142_{\pm .06}^{**}$	$0.775_{\pm .20}^{**}$	$0.666_{\pm .21}^{**}$	$0.422_{\pm .21}^{**}$
yolo_yolo11s-seg	$0.283_{\pm .26}^{**}$	$0.196_{\pm .21}^{**}$	$0.209_{\pm .15}^{**}$	$0.557_{\pm .40}^{**}$	$0.494_{\pm .40}^{**}$	$0.395_{\pm .23}^{**}$	$0.223_{\pm .25}^{**}$	$0.156_{\pm .23}^{**}$	$0.194_{\pm .22}^{**}$
yolo_yolo11m-seg	$0.330_{\pm .28}^{**}$	$0.234_{\pm .22}^{**}$	$0.241_{\pm .16}^{**}$	$0.589_{\pm .42}^{**}$	$0.540_{\pm .41}^{**}$	$0.507_{\pm .23}^{**}$	$0.214_{\pm .21}^{*}$	$0.140_{\pm .18}^{*}$	$0.192_{\pm .18}^{*}$
fastsam_FastSAM-x	$0.319_{\pm .20}^{**}$	$0.208_{\pm .16}^{**}$	$0.457_{\pm .11}^{**}$	$0.602_{\pm .21}^{**}$	$0.460_{\pm .21}^{**}$	$0.670_{\pm .09}^{**}$	$0.079_{\pm .12}^{**}$	$0.046_{\pm .08}^{**}$	$0.358_{\pm .13}^{**}$
ultra_sam_sam2.1_b	$0.234_{\pm .27}^{**}$	$0.165_{\pm .22}^{**}$	$0.145_{\pm .14}^{**}$	$0.499_{\pm .45}^{**}$	$0.467_{\pm .45}^{**}$	$0.392_{\pm .28}^{**}$	0.215_±.21_	0.140_±.17_	$0.231_{\pm .18}^{**}$
ultra_sam_sam2.1_l	$0.142_{\pm .18}^{**}$	$0.089_{\pm .13}^{**}$	$0.268_{\pm .15}^{**}$	$0.257_{\pm .29}^{**}$	$0.190_{\pm .26}^{**}$	$0.452_{\pm .20}^{**}$	0.285_±.31_	$0.218_{\pm .29}^{*}$	$0.458_{\pm .24}^{**}$

Higher is better. Values are mean_±SD_; bold marks the best mean per column. Superscripts denote the paired two-sided Wilcoxon signed-rank test versus ours_th68_fill (∗*p* < 0.05, ∗∗*p* < 0.01; no marker: not significant). SD is computed over images with a valid prediction; for methods with missing predictions, the evaluated count *n* differs (three-organ/soft-tissue *n* ≤ 8, 080, Kvasir *n* ≤ 590). MedSAM is evaluated with ground-truth-derived bounding-box prompts.

**Table 3:** Comparison of deformation region segmentation performance metrics (task-aligned segmentation baselines only)

Metric	TinySAM	SAM2	TEED	CFPNet-M	Ours
SSIM	0.3351	0.2977	0.0421	0.4525	**0.7660**
Accuracy	0.5656	0.5306	0.7044	0.0000	**0.9518**
Recall	0.0905	0.1721	0.3388	0.0000	**0.8346**
Precision	0.3256	0.3249	0.5772	0.4386	**0.9207**
F1 Score	0.1416	0.2250	0.4268	0.0000	**0.8758**

Bold values indicate the best performance for each metric.

**Table 4:** Boundary precision/recall/F1 (higher is better) on BSDS500 with binary edge maps

	BSDS500 train (200)			BSDS500 val (100)			BSDS500 test (200)		
Method	B-Prec *↑*	B-Rec *↑*	B-F1 *↑*	B-Prec *↑*	B-Rec *↑*	B-F1 *↑*	B-Prec *↑*	B-Rec *↑*	B-F1 *↑*
ours_th68	0.463_±.20_	0.358_±.15_	0.371_±.14_	0.466_±.21_	0.350_±.16_	0.367_±.14_	0.474_±.20_	0.344_±.13_	0.370_±.13_
ours_th90	$0.502_{\pm .21}^{**}$	$0.264_{\pm .13}^{**}$	$0.317_{\pm .14}^{**}$	$0.508_{\pm .22}^{**}$	$0.258_{\pm .15}^{**}$	$0.313_{\pm .15}^{**}$	$0.513_{\pm .22}^{**}$	$0.254_{\pm .12}^{**}$	$0.315_{\pm .13}^{**}$
ours_th95	$0.511_{\pm .21}^{**}$	$0.246_{\pm .13}^{**}$	$0.304_{\pm .14}^{**}$	$0.518_{\pm .22}^{**}$	$0.241_{\pm .15}^{**}$	$0.300_{\pm .15}^{**}$	$0.521_{\pm .22}^{**}$	$0.237_{\pm .12}^{**}$	$0.301_{\pm .13}^{**}$
sobel	$0.349_{\pm .17}^{**}$	$0.594_{\pm .13}^{**}$	$0.410_{\pm .14}^{**}$	$0.336_{\pm .16}^{**}$	$0.599_{\pm .13}^{**}$	$0.408_{\pm .14}^{**}$	$0.352_{\pm .16}^{**}$	$0.567_{\pm .13}^{**}$	$0.409_{\pm .13}^{**}$
laplacian	$0.315_{\pm .14}^{**}$	$0.610_{\pm .13}^{**}$	$0.397_{\pm .13}^{**}$	$0.307_{\pm .13}^{**}$	$0.615_{\pm .13}^{**}$	$0.396_{\pm .13}^{**}$	$0.316_{\pm .13}^{**}$	$0.585_{\pm .13}^{**}$	$0.394_{\pm .12}^{**}$
canny	$0.276_{\pm .15}^{**}$	$0.648_{\pm .13}^{**}$	$0.358_{\pm .12}^{*}$	$0.256_{\pm .14}^{**}$	$0.665_{\pm .13}^{**}$	0.342_±.12_	$0.271_{\pm .13}^{**}$	$0.647_{\pm .13}^{**}$	0.358_±.11_
dog	$0.313_{\pm .14}^{**}$	$0.612_{\pm .13}^{**}$	$0.396_{\pm .13}^{**}$	$0.306_{\pm .12}^{**}$	$0.610_{\pm .13}^{**}$	$0.395_{\pm .12}^{*}$	$0.313_{\pm .13}^{**}$	$0.584_{\pm .13}^{**}$	$0.393_{\pm .12}^{**}$
medsam_vit_b	–	–	–	0.435_±.12_	0.196_±.03_	0.270_±.05_	–	–	–
yolo_yolo11s-seg	$0.723_{\pm .18}^{**}$	$0.156_{\pm .12}^{**}$	$0.241_{\pm .16}^{**}$	$0.804_{\pm .17}^{**}$	$0.120_{\pm .10}^{**}$	$0.192_{\pm .15}^{**}$	$0.747_{\pm .18}^{**}$	$0.139_{\pm .09}^{**}$	$0.220_{\pm .13}^{**}$
yolo_yolo11m-seg	$0.791_{\pm .16}^{**}$	$0.163_{\pm .12}^{**}$	$0.250_{\pm .16}^{**}$	$0.838_{\pm .15}^{**}$	$0.119_{\pm .10}^{**}$	$0.190_{\pm .15}^{**}$	$0.773_{\pm .16}^{**}$	$0.139_{\pm .10}^{**}$	$0.221_{\pm .14}^{**}$
fastsam_FastSAM-x	$0.254_{\pm .23}^{**}$	$0.087_{\pm .09}^{**}$	$0.127_{\pm .13}^{**}$	$0.295_{\pm .22}^{**}$	$0.112_{\pm .10}^{**}$	$0.157_{\pm .13}^{**}$	$0.272_{\pm .20}^{**}$	$0.103_{\pm .10}^{**}$	$0.144_{\pm .13}^{**}$
ultra_sam_sam2.1_b	$0.525_{\pm .26}^{*}$	$0.074_{\pm .06}^{**}$	$0.120_{\pm .09}^{**}$	0.512_±.27_	$0.075_{\pm .06}^{**}$	$0.118_{\pm .09}^{**}$	$0.528_{\pm .25}^{*}$	$0.089_{\pm .07}^{**}$	$0.136_{\pm .10}^{**}$
ultra_sam_sam2.1_l	0.453_±.24_	$0.127_{\pm .10}^{**}$	$0.186_{\pm .13}^{**}$	0.497_±.26_	$0.127_{\pm .09}^{**}$	$0.185_{\pm .12}^{**}$	$0.530_{\pm .23}^{*}$	$0.146_{\pm .09}^{**}$	$0.213_{\pm .12}^{**}$

Values are mean_±SD_ over evaluated images (*n* = 200 train, 100 val, 200 test); bold marks the best mean per column. Superscripts denote the paired two-sided Wilcoxon signed-rank test versus ours_th68 (∗*p* < 0.05, ∗∗*p* < 0.01; no marker: not significant). Ground-truth boundaries are the union of all annotators. All methods are evaluated on binarized predictions. Tolerance is set to *t* = 1 pixel. If some predictions are missing, the evaluated image count may differ across methods.

**Table 5:** Boundary precision/recall/F1 (higher is better) on SBD with binary semantic boundary maps

	SBD train (8498)			SBD val (2857)		
Method	B-Prec *↑*	B-Rec *↑*	B-F1 *↑*	B-Prec *↑*	B-Rec *↑*	B-F1 *↑*
ours_th68	0.089_±.08_	0.340_±.19_	**0.127**_±.10_	0.091_±.08_	0.335_±.19_	0.127_±.10_
ours_th90	$0.097_{\pm .09}^{**}$	$0.255_{\pm .17}^{**}$	$0.124_{\pm .10}^{**}$	$0.099_{\pm .10}^{**}$	$0.249_{\pm .17}^{**}$	$0.123_{\pm .10}^{**}$
ours_th95	$0.099_{\pm .10}^{**}$	$0.237_{\pm .17}^{**}$	$0.123_{\pm .10}^{**}$	$0.101_{\pm .10}^{**}$	$0.232_{\pm .17}^{**}$	$0.121_{\pm .10}^{**}$
sobel	$0.067_{\pm .06}^{**}$	$0.520_{\pm .20}^{**}$	$0.111_{\pm .08}^{**}$	$0.067_{\pm .06}^{**}$	$0.520_{\pm .20}^{**}$	$0.111_{\pm .08}^{**}$
laplacian	$0.062_{\pm .05}^{**}$	$0.571_{\pm .19}^{**}$	$0.107_{\pm .07}^{**}$	$0.062_{\pm .05}^{**}$	$0.571_{\pm .20}^{**}$	$0.107_{\pm .08}^{**}$
canny	$0.052_{\pm .05}^{**}$	$0.588_{\pm .19}^{**}$	$0.089_{\pm .07}^{**}$	$0.052_{\pm .05}^{**}$	$0.587_{\pm .19}^{**}$	$0.089_{\pm .07}^{**}$
dog	$0.061_{\pm .05}^{**}$	$0.578_{\pm .19}^{**}$	$0.107_{\pm .07}^{**}$	$0.062_{\pm .05}^{**}$	$0.577_{\pm .19}^{**}$	$0.107_{\pm .07}^{**}$
medsam_vit_b	$0.039_{\pm .04}^{*}$	0.198_±.17_	$0.063_{\pm .07}^{*}$	$0.020_{\pm .01}^{**}$	$0.122_{\pm .08}^{*}$	$0.033_{\pm .02}^{**}$
fastsam_FastSAM-x	$0.073_{\pm .09}^{**}$	$0.130_{\pm .14}^{**}$	$0.086_{\pm .10}^{**}$	$0.074_{\pm .09}^{**}$	$0.134_{\pm .15}^{**}$	$0.087_{\pm .10}^{**}$

Values are mean_±SD_ over evaluated images (*n* = 8, 498 train, 2, 857 val); bold marks the best mean per column. Superscripts denote the paired two-sided Wilcoxon signed-rank test versus ours_th68 (∗*p* < 0.05, ∗∗*p* < 0.01; no marker: not significant). Ground-truth boundaries are computed as the union of class-wise GT boundaries. Tolerance is set to *t* = 1 pixel. If some predictions are missing (or heavy baselines are evaluated on a subset), the evaluated image count may differ across methods.

### Experimental design

Two sets of comparative analyses were performed. Experiment A focuses on contour localization and structural representation: we (1) compare against traditional and learning-based contour description/detection methods on endoscopic deformation regions and (2) verify robustness and universality across endoscopy, CT/MR, and natural images, reporting first-order and second-order contour variants of our method (Figure 5) together with quantitative results on public segmentation and boundary benchmarks (Tables 2, 3, 4, and 5). Experiment B targets deformation-aware detection/tracking on in-house endoscopic videos: we present qualitative comparisons and latent/mesh visualizations, and provide quantitative results using both pixel-level deformation-region segmentation metrics (Table 3) and counts against tracking/detection baselines, reflecting robustness under severe non-rigid deformation and appearance variation.

### Large-scale evaluation

We further validate scalability on the COCO-2017 benchmark with Train2017 (118K), Val2017 (5K), and Test2017 (20K) subsets. Adaptive deformation-based contour extraction is applied to generate paired training data, and performance on the test split is quantified using accuracy, precision, robustness, and generalization metrics, with comparisons against representative frameworks (e.g., YOLOv11, TinySAM, SAM2, TEED, and CFPNet-M).

### Reading guide and take-home messages

To facilitate interpretation of the diverse benchmarks, we connect the main quantitative comparisons (Tables 1, 2, 3, 4, and 5) with explicit take-home messages. These tables cover complementary tasks (mask segmentation vs. boundary/edge detection vs. deformation-region localization) and therefore report task-specific metrics (Dice/IoU/B-F1 and B-Prec/B-Rec/B-F1, plus SSIM and pixel-level metrics). All quantitative values are presented as mean_±SD_ over the per-image metric within each split; superscript markers in the tables denote the paired two-sided Wilcoxon signed-rank test between our method (ours_th68/ours_th68_fill) and each baseline (∗*p* < 0.05, ∗∗*p* < 0.01; no marker: not significant). Importantly, most public datasets rely on dense pixel-level human annotations (masks or 1-pixel boundaries); for deforming soft tissue, such labels are costly and can be ambiguous. Our primary goal is an interpretable, mathematically grounded deformation-cue extraction and implicit encoding that can also serve as a practical low-level annotation aid by producing stable contour/mask pseudo-labels for downstream matching/tracking and AR/MR navigation.

### Comparison with the contour description/detection methods

#### Evaluation of the proposed method in diverse scenarios

Contours serve as crucial visual cues for various applications, including contour-guided differentiable rendering. To comprehensively evaluate both performance and generalization, we tested our proposed method on natural images as well as MR/CT medical scans. Comparisons were conducted against several representative approaches: for natural scenes, large-scale models such as Segment Anything,^13^ SAM-Track, and YOLOv11; for medical data, U-Net variants, TEED,^16^ CaraNet,^20^ and Med-SAM.^17^^,^^18^ Figure 5 presents representative results: endoscopic images in Columns 1–3, MR/CT 4–5, and natural scene cases in Columns 6–7. Among the compared methods, YOLOv11 (Column 5) exhibits difficulty in achieving precise contour delineation, while other approaches (columns 2–4) often produce fragmented edges, missing regions, or false positive boundaries, particularly in cross-domain or low-contrast conditions. In contrast, our proposed method (columns 6–7) consistently generates smooth, continuous, and complete contours, highlighting its generalization ability, interpretability, and robustness across both natural and medical imaging domains. Overall, the results demonstrate that our method achieves higher accuracy and structural completeness compared with traditional and learning-based approaches across diverse visual conditions.

**Figure 6:**
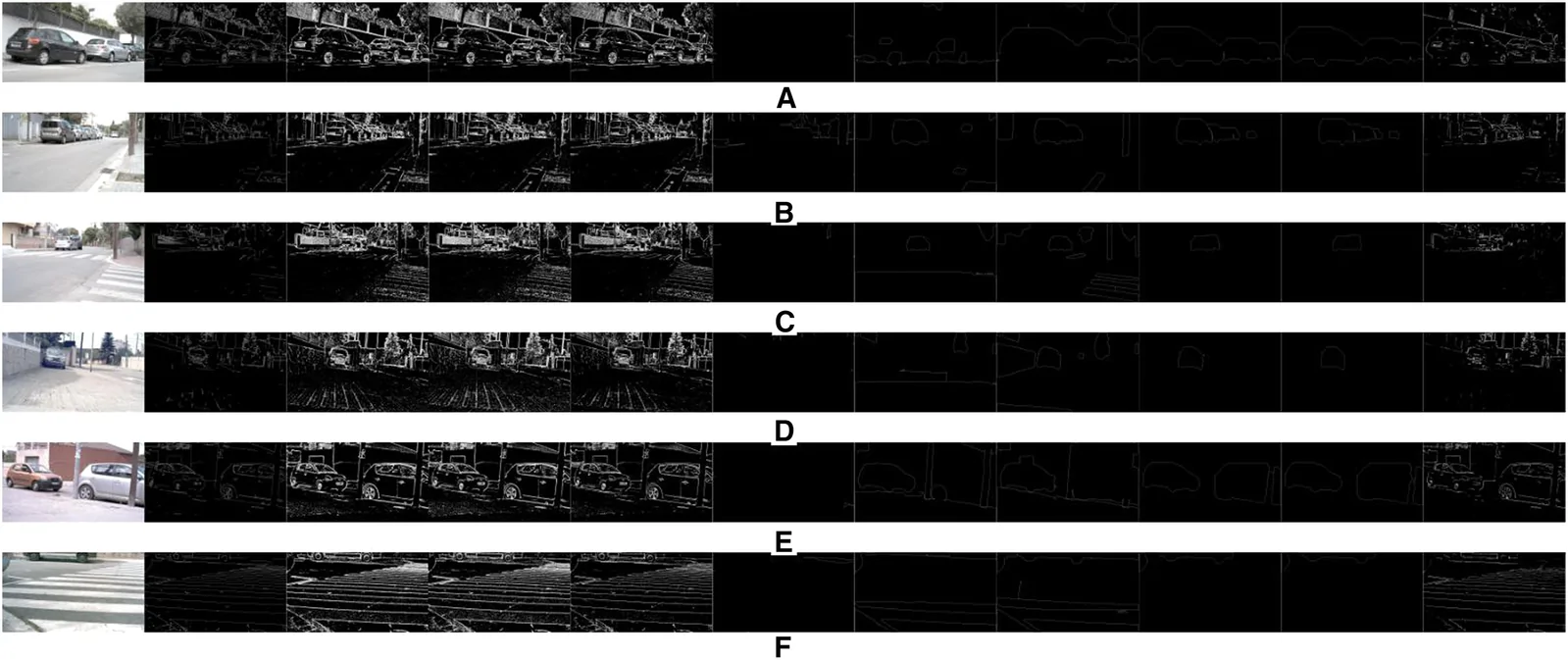
Qualitative comparison on BIPEDv2 The first column shows the input image. Columns 2–11 show the predicted 1-pixel boundaries from *Canny*, *DoG*, *Laplacian*, *Sobel*, *FastSAM*, *SAM2.1-b*, *SAM2.1-L*, *YOLO11m-seg*, and *YOLO11s-seg*, respectively. The last column shows the results of *Ours*. All predictions are binarized and visualized as boundary maps, consistent with the BIPEDv2 evaluation protocol (matching tolerance *t* = 1 pixel).

Table 1 reports contour/edge detection performance on BIPEDv2 using the standard split (200 train/50 test) with binary edge maps. Predictions are binarized and evaluated as 1-pixel boundaries under a matching tolerance of *t* = 1 pixel; we report B-Prec/B-Rec/B-F1 averaged over images. Classical gradient-based operators achieve substantially higher recall and the strongest overall B-F1 (e.g., Sobel), while our thresholded formulation favors higher precision at lower recall, with the threshold controlling this trade-off. Cross-task segmentation baselines (YOLO, FastSAM, and UltraSAM) perform poorly in this setting due to very low boundary recall after mask-to-boundary conversion. As illustrated in Figure 6, although our primary objective differs from generic edge benchmarks, qualitative visualizations indicate that our approach tends to recover more coherent and spatially consistent contours, often yielding larger and more connected contour regions.

**Figure 7:**
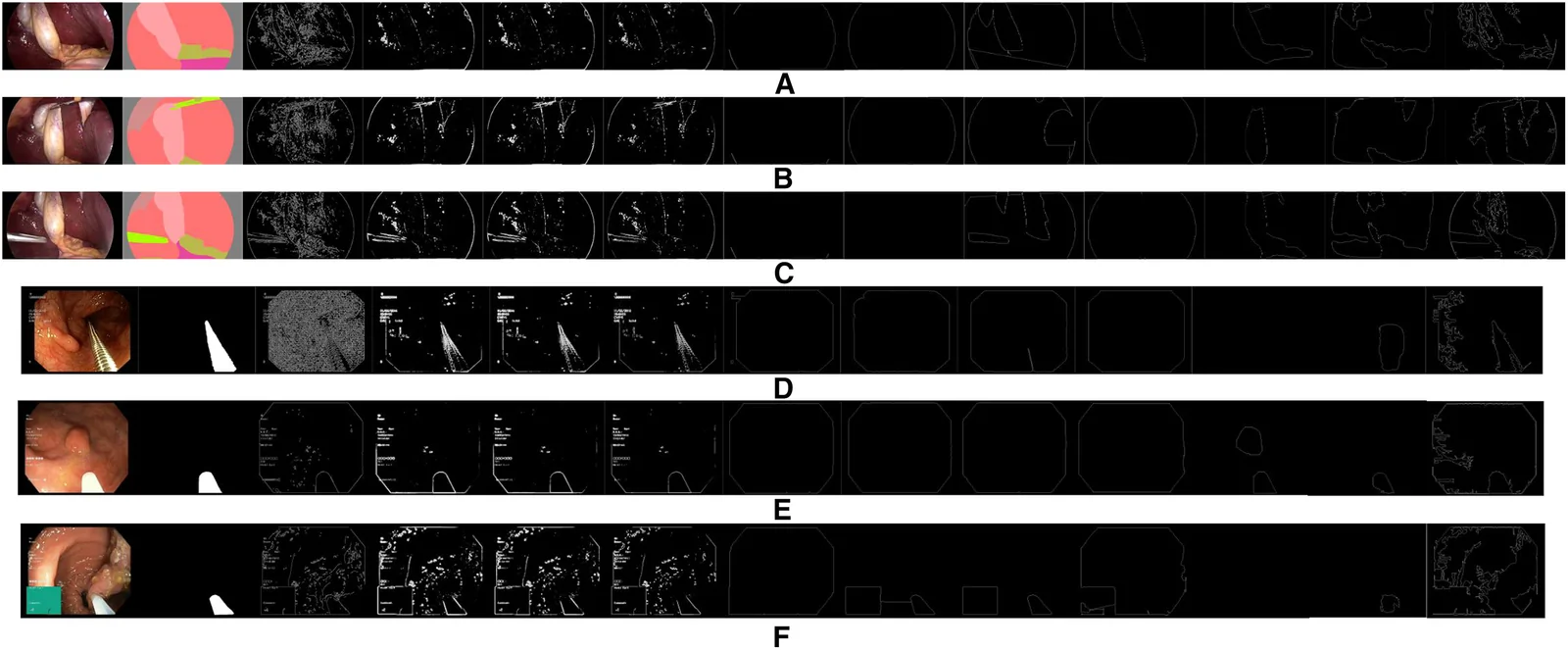
Qualitative comparison on CholecSeg8k and Kvasir-Instrument The first column shows the input image. Columns 3–12 show the predicted 1-pixel boundaries from *Canny*, *DoG*, *Laplacian*, *Sobel*, *FastSAM*, *SAM2.1-b*, *SAM2.1-L*, *YOLO11m-seg*, *YOLO11s-seg*, and *MedSAM*, respectively. The last column shows the results of *Ours*. All predictions are binarized and visualized as boundary maps, consistent with the CholecSeg8k and Kvasir-Instrument evaluation protocol (matching tolerance *t* = 1 pixel).

Table 2 (Mask segmentation on surgical datasets). We compare mask-producing methods under three binary segmentation settings: two binarized tasks on CholecSeg8k (archive; 8,080 frames), namely the three-organ union and the soft-tissue union, and the Kvasir-Instrument dataset (590 images) for instrument segmentation. We report Dice/IoU for region overlap and B-F1 for boundary agreement (after converting masks to 1-pixel boundaries) under the same evaluation protocol, with scores averaged over images. In addition to general-purpose segmentation baselines (MedSAM, YOLO, FastSAM, and UltraSAM), we include our contour-filling variants at multiple thresholds (th68/th90/th95) to characterize the trade-off between region coverage and boundary agreement. MedSAM (with ground-truth-derived box prompts) achieves the best Dice/IoU on the three-organ and Kvasir settings, while our th68 filling variant yields the best Dice/IoU on the soft-tissue setting; in terms of boundary agreement, FastSAM attains the highest B-F1 on the two CholecSeg8k settings, and UltraSAM-l performs best on Kvasir. As further illustrated by the qualitative comparison in Figure 7, although these datasets target different objectives, qualitative visualizations consistently indicate that our approach tends to recover larger and more connected contour regions.

**Figure 8:**
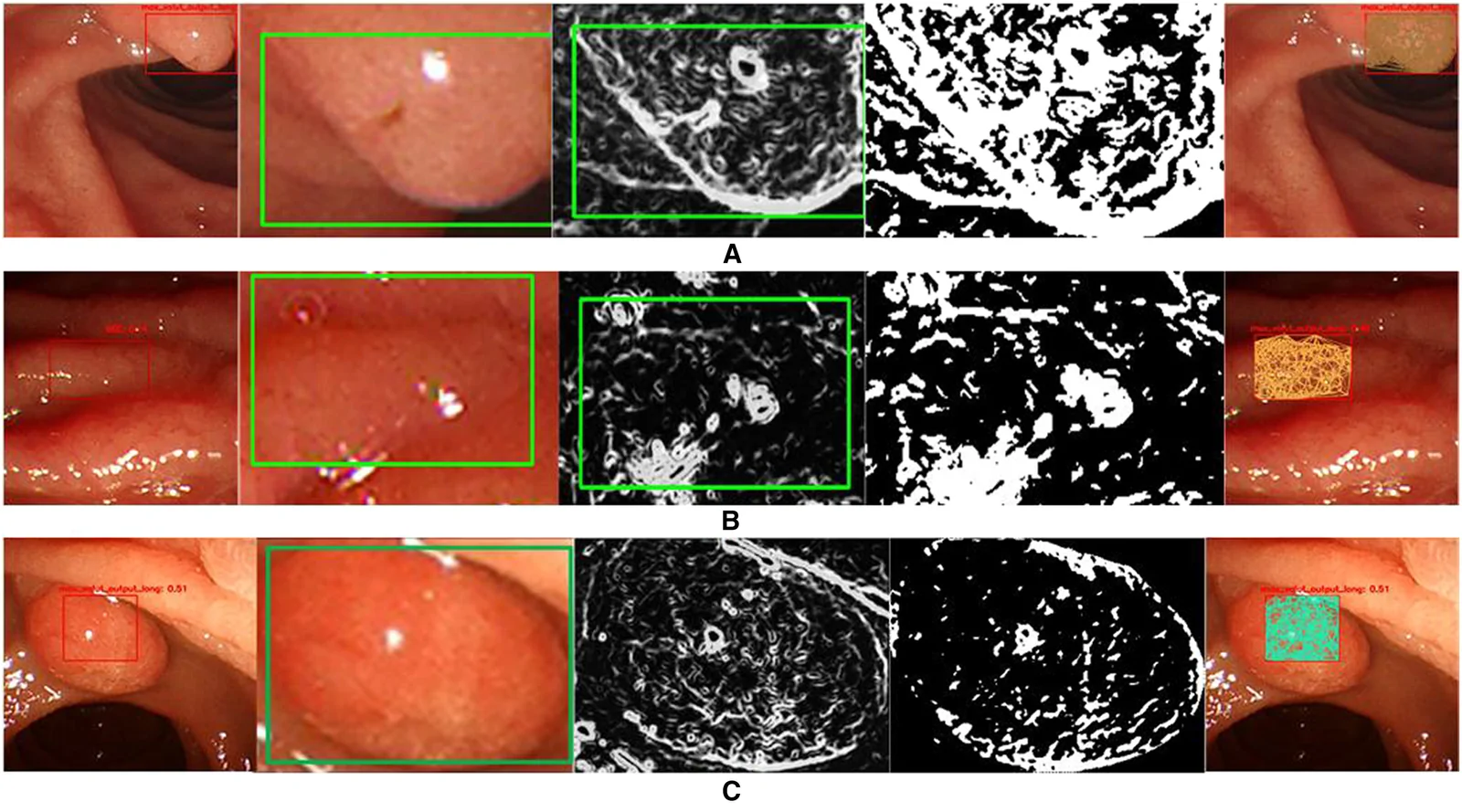
Endoscopic deformation-contour detection and latent-code visualization From left to right: original frames with annotated deformation regions, magnified deformation areas, corresponding detection outputs, visualization of the latent representations learned by our encoder-decoder (implicit and semantic codes), and a mesh-based visualization for deformation-region detection and tracking, illustrating the potential of the proposed method in deformation-aware tracking scenarios.

Table 3 presents a quantitative assessment of deformation region segmentation. We report SSIM and standard pixel-level segmentation metrics (accuracy, recall, precision, and F1 score) computed against manually annotated deformation masks. The annotations correspond to the deformation regions shown in Figure 8 (first column) and are used as pixel-level reference masks. SSIM measures structural similarity between predicted deformation contours/masks and the annotations, while accuracy, precision, recall, and F1 score quantify pixel-level agreement between predictions and annotations. As summarized in Table 3, our method achieves the best overall performance among the compared segmentation baselines. Following the reviewers’ suggestions, detection-only baselines are used for qualitative comparison and are not included in this pixel-level quantitative table.

**Figure 9:**
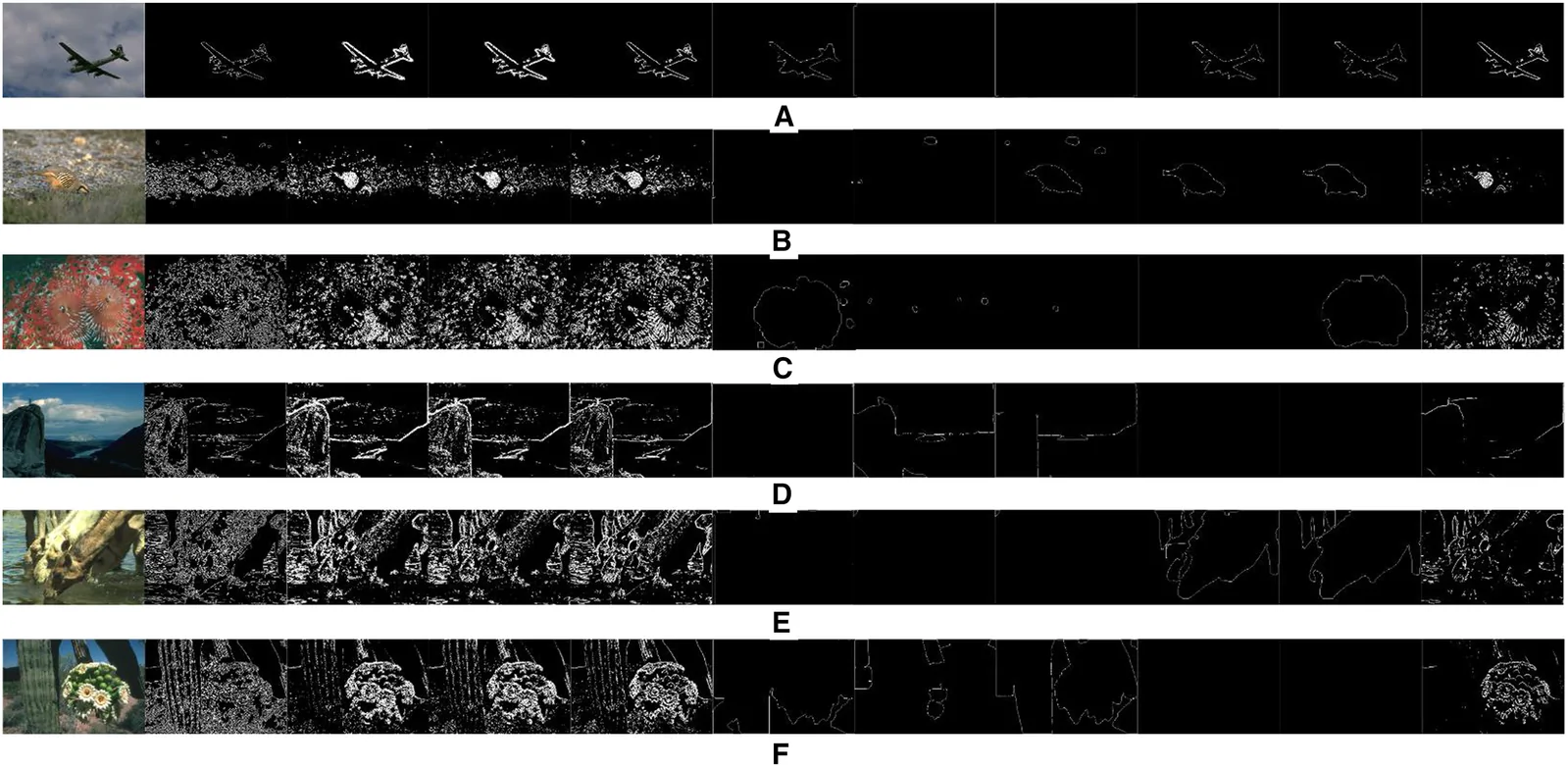
Qualitative comparison on BSDS500 The first column shows the input image. Columns 2–11 show the predicted 1-pixel boundaries from *Canny*, *DoG*, *Laplacian*, *Sobel*, *FastSAM*, *SAM2.1-b*, *SAM2.1-L*, *YOLO11m-seg*, and *YOLO11s-seg*, respectively. The last column shows the results of *Ours*. All predictions are binarized and visualized as boundary maps, consistent with the BSDS500 evaluation protocol (matching tolerance *t* = 1 pixel).

Table 4 summarizes generic contour/edge detection results on BSDS500 using the official split (200/100/200 train/val/test). BSDS500 provides multi-annotator boundary annotations; we form a binary ground-truth edge map by taking the union of all annotators and evaluate all methods on binarized boundary predictions (segmentation-based baselines are converted from masks to 1-pixel boundaries). We report B-Prec/B-Rec/B-F1 with a matching tolerance of *t* = 1 pixel and average scores over images (with method-specific counts when predictions are missing). Classical operators generally achieve higher recall; Sobel attains the best B-F1 across splits, while Canny yields the highest B-Rec. Our thresholded formulation does not achieve the top score on BSDS500, but it is stable across train/val/test and provides a controllable trade-off via the threshold. As illustrated in Figure 9, although our primary target differs from generic edge benchmarks, qualitative visualizations of the produced contours indicate that our approach often recovers more accurate and more complete contour information, yielding larger and more connected contour regions.

**Figure 10:**
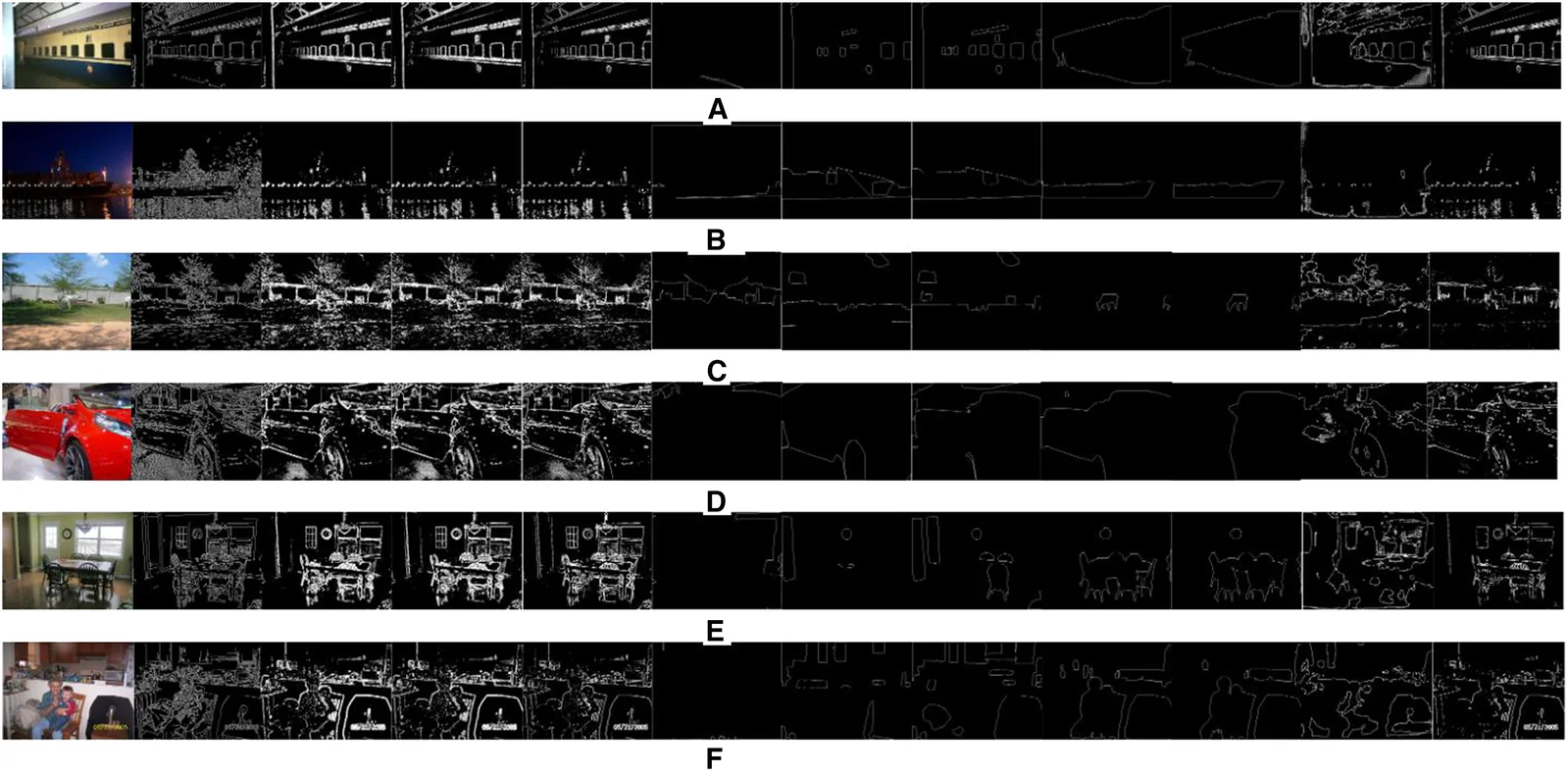
Qualitative comparison on SBD The first column shows the input image. Columns 2–12 show the predicted 1-pixel boundaries from *Canny*, *DoG*, *Laplacian*, *Sobel*, *FastSAM*, *SAM2.1-b*, *SAM2.1-L*, *YOLO11m-seg*, *YOLO11s-seg*, and *MedSAM*, respectively. The last column shows the results of *Ours*. All predictions are binarized and visualized as boundary maps, consistent with the semantic-boundary evaluation protocol (multi-annotator union GT; matching tolerance *t* = 1 pixel).

Table 5 reports semantic boundary detection results on the Semantic Boundaries Dataset (SBD; 8,498 training and 2,857 validation images). Using the official GTcls annotations, we construct a binary ground-truth boundary map per image by taking the union of class-wise boundaries for the categories present. All methods are evaluated on binary 1-pixel boundary predictions (segmentation outputs are converted from masks to boundaries) using B-Prec/B-Rec/B-F1 with a matching tolerance of *t* = 1 pixel; scores are averaged over images (with method-specific counts when some predictions are missing). This protocol enables task-consistent comparison of boundary localization and coverage: classical edge operators often favor recall at the expense of precision, whereas our thresholded formulation provides a controllable balance and behaves consistently across splits. As illustrated in Figure 10, although the datasets in our study target different objectives, qualitative visualizations indicate that our approach tends to recover more complete and connected contour regions.

Summary of key quantitative findings (Tables 1, 2, 3, 4, and 5). We highlight concise per-table take-home messages:

• Table 1 **(BIPEDv2 boundary/edge):** Classical gradient operators achieve substantially higher recall and the strongest B-F1 (e.g., Sobel), whereas our thresholded formulation favors higher precision with a controllable precision-recall trade-off and yields more coherent/connected contours in qualitative visualizations.
• Table 2 **(CholecSeg8k & Kvasir-Instrument mask segmentation):** Under the soft-tissue union setting, our contour-filling variant achieves the best Dice/IoU, while MedSAM (with ground-truth-derived box prompts) performs best on the three-organ union and instrument settings; boundary agreement (B-F1) varies by dataset and method.
• Table 3 **(Deformation-region segmentation):** Against manual deformation masks on three endoscopic sequences, our method achieves the best overall SSIM and pixel-level metrics (accuracy/precision/recall/F1) among task-aligned segmentation baselines.
• Table 4 **(BSDS500 boundary/edge):** On generic edge benchmarks, classical operators dominate recall and B-F1, while our method remains stable across splits and provides a tunable threshold trade-off, reflecting our different design goal (interpretable deformation-contour coherence rather than generic edge coverage).
• Table 5 **(SBD semantic boundaries):** Despite the semantic boundary setting, our method achieves the best B-F1 among the compared methods and behaves consistently across splits, supporting robustness beyond the endoscopic domain.

### Detection/tracking evaluation on a standard controllable dataset

#### Comparison with the target detection/tracking methods

To comprehensively evaluate the potential application, we qualitatively and quantitatively compare the proposed approach with the existing target tracking/detection methods. We evaluate tracking performance on a standardized chessboard dataset comprising 11 video sequences (3,848 frames total) captured under controlled imaging conditions with varying illumination, occlusion, and specular reflections. Per-frame ground-truth bounding boxes and binary masks are generated automatically through chessboard corner detection with sub-pixel precision.

#### Dataset construction rationale

Existing endoscopic tracking datasets, including TrackVes, CholecSeg8k, SurgT-benchmarking, and EndoVis Instrument Tracking, rely on manual or semi-automatic annotation, which introduces subjectivity and potential inaccuracies in ground-truth labels. Boundary definitions for deformable surgical targets under specular reflections, smoke, and fluid occlusion remain ambiguous, making quantitative comparisons between methods difficult to interpret. To address these limitations, we construct a standardized, controllable evaluation dataset using chessboard markers with three different specifications: 11 × 8 squares (11 mm per square), 9 × 8 squares (8 mm), and 9 × 6 squares (5 mm). The chessboard serves as a ground-truth reference with several advantages:

Pixel-accurate ground truth: Corner detection algorithms localize chessboard vertices with sub-pixel precision, enabling automatic generation of bounding boxes and binary masks without manual annotation. The geometric constraints of the chessboard pattern provide inherent validation of annotation correctness. Controlled challenge variations: By capturing sequences under different conditions, we systematically introduce tracking challenges including rotation (target articulation), translation (camera motion), scale changes (zoom, depth variation), partial occlusion (object collisions), and illumination changes (specular highlights, shadow casting). Reproducibility: The dataset construction pipeline is fully automated, from image capture through chessboard detection to ground-truth export, ensuring consistency across all 11 sequences (3,848 frames total) and enabling other researchers to replicate the evaluation protocol. This controlled evaluation framework allows us to assess tracking methods under known ground-truth conditions, providing objective measurements of accuracy, precision, and robustness across varying challenge profiles.

#### Experimental setup

The dataset comprises 11 video sequences (3,848 frames total) captured under controlled conditions with systematic variations in illumination, occlusion, and camera viewpoint. Three chessboard specifications are used to evaluate scale robustness: 11 × 8 squares (11 mm per square, 3 sequences), 9 × 8 squares (8 mm, 3 sequences), and 9 × 6 squares (5 mm, 5 sequences). Each chessboard is mounted on a moving target and undergoes rotation, translation, scale changes, and partial occlusion during capture. Per-frame ground-truth bounding boxes and binary masks are generated automatically through chessboard corner detection with sub-pixel precision.

We compare our tracker against representative methods from different methodological categories: CSRT^44^ (correlation-filter tracker), SAM^13^ (foundation model), and YOLO-Seg^45^ (one-stage segmentation). Transformer-based methods (OSTrack,^40^ MixFormer,^41^ LMTrack,^42^ Cutie^43^) were evaluated with randomly initialized backbones due to offline test environment constraints, serving as architecture reference baselines.

Evaluation follows standard tracking metrics: coverage-aware BBox IoU, Precision Rate, Success Rate, and Center Error. The coverage-aware IoU assigns a score of 1.0 when the prediction fully contains the ground-truth region, reflecting the requirement for complete target visibility. Precision rate measures the proportion of frames with center point error ≤20 pixels. Success Rate measures the proportion of frames with IoU ≥0.5. Center Error computes the Euclidean distance between predicted and ground-truth center points in pixels.

Table 6 presents the comparison results across all 11 sequences under the unified coverage-aware BBox IoU protocol. Our tracker attains a mean IoU of 0.809 ± 0.211, together with the highest success rate (0.888), precision rate (0.648), and the lowest center error (70.8 px). The paired two-sided Wilcoxon signed-rank test indicates that our method achieves a significantly higher per-sequence IoU than every baseline (*p* < 0.01 in all cases). Among the randomly initialized transformer-based methods, MixFormer attains the highest baseline IoU (0.540) owing to the strong inductive bias of self-attention for template matching, followed by LMTrack (0.536) and OSTrack (0.523). SAM reaches 0.333 IoU but exhibits larger center drift, while YOLO-seg (0.305) and Cutie (0.288) are limited by single-frame detection and memory-based propagation, respectively, under rapid appearance changes.

**Table 6:** Tracker performance on the standardized chessboard dataset (11 sequences, 3,848 frames), evaluated under a unified coverage-aware BBox IoU protocol

Method	IoU *↑*	Prec. *↑*	Succ. *↑*	Ctr.Err. *↓*	Notes
**Ours**	**0.809**_±0.21_	**0.648**	**0.888**	**70.8**	Scheme C + NanoTrack
MixFormer^41^	$0.540_{\pm 0.11}^{**}$	0.019	0.440	217.6	ViT (rand.)
LMTrack^42^	$0.536_{\pm 0.18}^{**}$	0.017	0.444	219.1	ViT (rand.)
OSTrack^40^	$0.523_{\pm 0.13}^{**}$	0.014	0.425	225.9	ViT (rand.)
CSRT^44^	$0.356_{\pm 0.33}^{**}$	0.129	0.291	231.5	correlation filter
SAM^13^	$0.333_{\pm 0.27}^{**}$	0.226	0.323	341.4	foundation model
YOLO-seg	$0.305_{\pm 0.17}^{**}$	0.091	0.301	544.0	one-stage
Cutie^43^	$0.288_{\pm 0.22}^{**}$	0.049	0.219	237.0	VOS (rand.)

IoU is reported as mean_±SD_ over the 11 sequences; bold marks the best value per column. Superscripts denote the paired two-sided Wilcoxon signed-rank test of our method versus each baseline on the per-sequence IoU (∗*p* < 0.05, ∗∗*p* < 0.01).

Coverage-aware IoU: a prediction fully covering the ground-truth box scores 1.0; otherwise, the standard Jaccard index is used. Transformer trackers (OSTrack, MixFormer, LMTrack, Cutie) use randomly initialized backbones (architecture reference only, offline setting).

The proposed tracker builds on ContourSimilarityNet (pre-trained on the chess v1 dataset with RTW augmentation, 173 template views, and th68 binarization) and augments it with a hierarchical multi-feature (contour, color-histogram, semantic) matching scheme and a lightweight NanoTrack-based re-detection module that recovers the target when the contour confidence drops. The learned contour similarity provides a consistent shape-correspondence signal (0.9925 mean similarity across all sequences), and an early-exit mechanism (threshold 0.95) reduces computation on high-confidence frames at 0.25 resolution scale.

#### Validation of the deformation detection method in the tracking process

Traditional learning-based tracking methods typically use bounding boxes to represent targets. Our approach, however, captures the key deformation features of soft tissue surfaces directly. Figure 8 demonstrates the performance of the proposed method for detecting and representing soft tissue deformations in endoscopic video sequences (Video S1). The annotated regions in the first column serve as references for ground truth deformations. The following columns show the magnified regions, highlighting the model’s ability to accurately localize and describe subtle tissue deformations. The latent encoding maps visualize high-level semantic representations extracted by our framework, revealing consistent contour and texture information. Finally, the mesh-based visualization provides an intuitive depiction of the deformation structure and its spatial continuity, further confirming the robustness and precision of our method in tracking non-rigid soft tissue motions.

Video S1. Demonstration of soft-tissue surface deformation detection and tracking in an endoscopic video sequence

## Discussion

This study introduces a unified framework for contour-based description, semantic representation, and graph-guided detection of soft tissue surface deformations in digestive endoscopy. The proposed method adaptively localizes deformation regions and addresses the sensitivity of existing descriptors to noise. Compared with learning-based contour detection approaches, our method is both interpretable and more effective. The semantic descriptor is applicable to AR/MR surgical navigation and provides stable features for 2D/3D reconstruction. Experimental results demonstrate robustness and semantic accuracy in contour-based detection and characterization of deformable soft tissue regions.

### Limitations of the study

A current limitation lies in handling extreme deformation, which may introduce local contour discontinuities in flattened areas. Nonetheless, these do not significantly affect feature extraction or correspondence accuracy. We also note that computational latency and real-time feasibility have not yet been systematically optimized, and performance may degrade under abrupt camera motion or zoom changes. In future work, we will benchmark runtime and memory usage and improve efficiency for real-time navigation. Finally, while we observe promising generalization across the datasets tested, broader validation on other organs and surgical scenarios remains necessary. Because only fully de-identified video data were available to the research team, the potential influence of participant sex, gender, age, or other demographic characteristics could not be assessed.

## Resource availability

### Lead contact

Requests for further information and resources should be directed to and will be fulfilled by the lead contact, Ranyang Li (lry@haut.edu.cn).

### Materials availability

This study did not generate new materials.

### Data and code availability

• All data reported in this paper will be shared by the lead contact upon reasonable request. Access to certain clinical data may be subject to institutional agreements and privacy regulations.
• This paper reports original code, which is publicly available on GitHub: https://github.com/liranyang123456-commits/Soft-Tissue-Surface-Deformation-Detection-in-Endoscopic-Images. The repository link is also listed in the key resources table.
• Any additional information required to reanalyze the data reported in this paper is available from the lead contact upon reasonable request.

## Acknowledgments

This research is supported by 10.13039/501100001809National Natural Science Foundation of China (6240074117); 10.13039/501100003489Henan University of Technology Young Backbone Teachers Training Program (no. 21421329); Open Project Program of 10.13039/501100011160State Key Laboratory of Virtual Reality Technology and Systems (VRLAB2023C03); the high-level Talents Fund of 10.13039/501100003489Henan University of Technology (nos 2022BS076 and 2022BS075) and Cultivation Project of Tuoxin Team in 10.13039/501100003489Henan University of Technology (2024TXTD18).

## Author contributions

Conceptualization, R.L. and N.W.; methodology, W.L., R.L., and N.W.; investigation, X.G., C.F., R.L., and N.W.; writing – original draft, R.L. and Z.Z.; writing – review and editing, R.L. and J.P.; funding acquisition, R.L. and N.W.; resources, R.L. and X.G.; supervision, R.L. and J.P.

## Declaration of interests

The authors declare no competing interests. Author Junjun Pan is a Guest Editor on the special issue “Multimodal Image Analysis, Navigation, and Robotics in Medicine” published in iScience.

## Declaration of generative AI and AI-assisted technologies in the writing process

The authors declare that no generative AI or AI-assisted technologies were used in the creation of this manuscript.

## STAR★Methods

### Key resources table

**Table:** 

REAGENT or RESOURCE	SOURCE	IDENTIFIER
**Software and algorithms**		

Python	Python Software Foundation	https://www.python.org/
JetBrains IDEs	PyCharm	https://www.jetbrains.com.cn/en-us/pycharm/
OpenCV	Open Source Computer Vision Library	https://opencv.org/
Original code (this paper)	This paper; GitHub	https://github.com/liranyang123456-commits/Soft-Tissue-Surface-Deformation-Detection-in-Endoscopic-Images

### Experimental model and study participant details

#### Human participant data and ethics

This study was a retrospective secondary analysis of fully de-identified intraoperative endoscopy and laparoscopy videos supplied by Henan Provincial People’s Hospital through collaborating clinician Nan Wei, M.D. The videos were de-identified by the hospital in accordance with its data-handling requirements before transfer to the research team. The work involved no participant recruitment, clinical intervention, or direct contact with patients; the authors received video files only and had no access to identifiable personal information or other participant-level records. The use of these de-identified data was conducted in accordance with the hospital’s requirements.

#### Participant characteristics and experimental models

Because the research team received only fully de-identified video data, participant-level information, including age, sex, gender, ancestry, race, and ethnicity, was unavailable. Consequently, the association of these characteristics with the results could not be assessed, which limits the generalizability of the findings. No animal experiments, animal-derived specimens, cell lines, primary cell cultures, or *ex vivo* tissue samples were used in this study.

#### Dataset summary

The medical subset includes existing intraoperative digestive endoscopy and laparoscopic cholecystectomy videos covering three representative soft-tissue regions (duodenal papilla, peristaltic intestine, and planar mucosal folds). The three representative endoscopic sequences used in quantitative evaluations contain 128, 164, and 206 frames, respectively. These sequences were used only for algorithmic evaluation; no experimental allocation of participants or interventions was performed. Natural-scene sequences and CT/MRI volumes were additionally used to assess cross-domain generalization.

### Method details

The limited field of view in digestive endoscopic images, combined with the smooth and highly deformable nature of soft tissue surfaces, poses significant challenges for existing contour description and tracking methods. To address these issues, we propose a framework for deformation detection and tracking, as illustrated in Figure 2. First, we introduce an adaptive deformation detection strategy that identifies image contours and local deformation regions by computing inflection points in the information density distribution function within the gradient domain. This approach enables precise and data-driven extraction of soft tissue surface deformations, providing reliable cues for downstream representation and tracking. To improve computational efficiency and reduce the runtime of inflection point detection, we design a universal, multi-task, end-to-end network that integrates self-attention mechanisms, residual connections, and multi-layer perceptrons (MLPs). This architecture allows for simultaneous extraction of deformation regions and their semantic encodings, achieving both accuracy and efficiency in representing complex, non-rigid tissue motions. Finally, by fusing deformation contours, semantic latent encodings, and color features within a spatiotemporal context module, the proposed framework enables robust and temporally coherent tracking of soft tissue surface deformations in endoscopic imagery. This integrated approach directly addresses the challenges of limited field of view, subtle non-rigid deformations, and low-texture regions, supporting feature matching and registration in AR/MR navigation.

#### Deformation detection in soft tissue surface

Traditional model-based methods are highly susceptible to noise interference, whereas deep learning and large-scale vision models rely heavily on large quantities of manually annotated data. However, due to the inherent uncertainty and subjectivity of human visual judgment, pixel-level contour or edge annotations often lack precision, resulting in inconsistent or ambiguous outcomes in complex imaging environments. To overcome these limitations and achieve accurate detection and tracking of soft tissue surface deformations in endoscopic imagery, we propose a new probabilistic deformation detection framework. By formulating a natural image *I*(*x*, *y*) as a Gaussian random field, we theoretically establish that the probability density function of the first-order gradient, after applying Gaussian smoothing, follows a non-standard Gaussian distribution. This provides a statistical basis for deformation-sensitive contour extraction. The mathematical derivation proceeds as follows: Let *I*(*x*, *y*) denote the input image, and define the horizontal gradient at pixel (*i*, *j*) as the convolution between the image and the first-order derivative of a Gaussian kernel:

(Equation 1) $$\begin{array}{c} I=\overset{M}{\underset{i=1}{\sum }}E_{i}+\overset{N}{\underset{j=1}{\sum }}R_{j}+\overset{S}{\underset{k=1}{\sum }}B_{k},\mu =\frac{1}{M+N+S}\left(\overset{M}{\underset{i=1}{\sum }}E_{i}+\overset{N}{\underset{j=1}{\sum }}R_{j}+\overset{S}{\underset{k=1}{\sum }}B_{k}\right)\\ G_{x}\left[i,j\right]=\left(\frac{\partial G_{\sigma }}{\partial x}*I\right)\left[i,j\right]=\underset{u,v}{\sum }w_{u,v}\;I\left[i-u,j-v\right]. \end{array}$$

(Equation 2) $$\begin{array}{cc} w_{u,v} & =\frac{\partial G_{\sigma }}{\partial x}\left(u,v\right)=-\frac{u}{\sigma^{2}}\;G_{\sigma }\left(u,v\right),\\ G_{\sigma }\left(u,v\right) & =\frac{1}{2\pi \sigma^{2}}\exp\;\left(-\frac{u^{2}+v^{2}}{2\sigma^{2}}\right). \end{array}$$

Generally, the information in the gradient space can be decomposed into three signal components, expressed as where **E** denotes contour-related features with strong gradient responses, **R** represents high-frequency components such as noise or specular reflections, and **B** corresponds to low-frequency regions characterized by weak gradients. The gradient information matrix **I** can be approximated by a Gaussian distribution, where *u* and *σ* denote the mean and variance of gradient intensities, respectively: $N\left(u,\sigma^{2}\right)$. According to the properties of the Gaussian distribution, the inflection points of the probability density curve occur at *u* ± *σ*. Therefore, by analyzing the gradient density distribution around these inflection points, we can accurately identify and localize deformation regions on soft tissue surfaces. Assume the local pixels *X*_*u*,*v*_ = *I*[*i* − *u*, *j* − *v*] are identically distributed, weakly correlated random variables with $E\left[X_{u,v}\right]=\mu$ and $Var\left(X_{u,v}\right)=\sigma_{I}^{2}$. Define *S*_*N*_ = *∑*_*u*,*v*_*w*_*u*,*v*_ *X*_*u*,*v*_, since *∑*_*u*,*v*_*w*_*u*,*v*_ = 0, In what follows, we denote the weighted sum of local pixel values by *S*_*N*_ = *∑*_*u*,*v*_*w*_*u*,*v*_*X*_*u*,*v*_, where $w_{u,v}=\frac{\partial G_{\sigma }}{\partial x}\left(u,v\right)$ and *X*_*u*,*v*_ = *I*[*i* − *u*, *j* − *v*].

Therefore, its distribution follows:

(Equation 3) $$E\left[S_{N}\right]=u,\;Var\left(S_{N}\right)=\sigma_{I}^{2}\underset{u,v}{\sum }w_{u,v}^{2}.$$

(Equation 4) $$\begin{array}{cc} \varphi_{S_{N}}\left(t\right) & =\underset{u,v}{\prod }E\left[e^{i\;t\;w_{u,v}X_{u,v}}\right]\approx \exp\;\left(-\frac{1}{2}\;t^{2}\;\sigma_{I}^{2}\underset{u,v}{\sum }w_{u,v}^{2}\right),\\ S_{N} & \overset{d}{\to }N\;\left(u,\;\sigma_{I}^{2}\underset{u,v}{\sum }w_{u,v}^{2}\right), \end{array}$$

which is exactly that of a Gaussian distribution with mean *u* and variance $\sigma_{I}^{2}\sum_{u,v}w_{u,v}^{2}$. By the Lindeberg–Feller central limit theorem. So the first-order Gaussian-smoothed gradient *G*_*x*_[*i*, *j*] is approximately Gaussian.

Leveraging the properties of the Gaussian distribution and the characteristics of first-order gradient images, it can be seen that most gradient values fall within the range [*μ* − *δ*, *μ* + *δ*], covering approximately 68.27% of the data. These represent low-frequency, “stable” features, while high-frequency “salient” features correspond to significant gradient variations outside this interval. Let *F*(*x*) denote the cumulative histogram percentage at intensity *x*. Then the threshold for percentile *α* is given by *t*_*α*_ = min{*x*∣*F*(*x*) ≥ *α*}, for example *t*_0.68_ = min{*x*∣*F*(*x*) ≥ 0.68}. As shown in Figure 4, experimental results further validate this observation under different statistical thresholds—namely, the median, mean, 68%, and 95% confidence levels. When using the median and mean thresholds, the detected deformation regions appear incomplete and less accurate, failing to capture subtle yet significant contour changes. In contrast, the 95% threshold yields overly conservative results, identifying only a few deformation areas and thus underrepresenting the actual deformation distribution. These findings indicate that the gradient-based deformation detection is highly sensitive to the choice of threshold, and a balance between precision and completeness must be carefully maintained.

#### Deformation region semantic coding generation

Accurate detection and extraction of soft tissue deformation regions demand precise localization, characterization, and quantitative assessment of surface shape variations. Traditional contour-based approaches mainly depend on low and mid-level representations such as spatial transform features (e.g., FFT, wavelet, Gabor), geometric descriptors, and distance-driven similarity metrics. However, deformable contours often involve dense pixel regions, resulting in heavy computational loads and prolonged processing time. In addition, handcrafted features are not easily compatible with deep learning architectures, creating integration challenges and limiting scalability. More importantly, they lack high-level semantic awareness, which constrains both accuracy and robustness under complex surgical scenes. To overcome these drawbacks, we introduce a generalized, multi-task, end-to-end deformation-aware network specifically designed for large-scale medical imagery. The proposed framework efficiently models soft tissue deformation in minimally invasive endoscopic procedures by leveraging self-attention, residual learning, and a symmetric encoder–decoder topology. As shown in Figures 2 and 3, the network adopts a U-shaped symmetric architecture comprising three main modules: an encoder, a bottleneck, and a decoder. Multi-level skip connections facilitate fusion of low-level structural cues and high-level semantic features. The encoder is composed of five residual stages, each containing two 3 × 3 convolutional layers followed by batch normalization (BN) and ReLU activation. The input RGB image is first processed by an initial convolutional layer and subsequently passed through residual blocks with increasing channel dimensions of 64, 128, 256, 512, and 1024. Downsampling is achieved through 2 × 2 max-pooling. To better capture non-rigid motion patterns, a multi-head self-attention module is embedded after each residual stage, enabling long-range spatial dependency modeling and enriched contextual representation.

The input image is denoted by $I\in R^{3\times H\times W}$, and the output is the predicted deformation region map $\hat{Y}\in R^{3\times H\times W}$. The network consists of a symmetric encoder-decoder structure. In each encoder layer, two consecutive convolution operations are applied, described as:

(Equation 5) $$F^{\left(l\right)}=ReLU\left(BN\left(W^{\left(l\right)}*F^{\left(l-1\right)}+b^{\left(l\right)}\right)\right).$$

where **W**^(*l*)^ and **b**^(*l*)^ are the convolution kernel and bias at layer *l*, respectively, and **F**^(0)^ = **I**. BN denotes batch normalization, and ReLU is the activation function.

At the core of the network lies the bottleneck module, positioned at the deepest layer of the architecture. It is composed of multiple deep residual convolutional units designed to aggregate and refine high-level semantic representations extracted by the encoder. The incorporation of self-attention mechanisms further strengthens the model’s capacity to distinguish subtle deformation cues and to encode more abstract, context-aware feature embeddings. The decoder branch is constructed symmetrically to the encoder, containing five progressive upsampling stages. Each stage restores spatial resolution through either transposed convolution or bilinear interpolation followed by convolutional refinement, and includes two 3 × 3 convolutional layers with batch normalization and ReLU activation. To reconstruct detailed edge and contour structures, skip connections are established between each encoder–decoder pair, enabling direct information flow from shallow to deep layers. Additionally, self-attention modules embedded within the decoder enhance the model’s ability to focus on crucial regions, such as deformation boundaries. Finally, a 1 × 1 convolution layer with Sigmoid activation outputs a pixel-wise deformation probability map or a semantic mask representing the detected deformation areas.

After convolution, spatial downsampling is performed via max pooling:

(Equation 6) $$F_{\text{down}}^{\left(l\right)}=MaxPool\left(F^{\left(l\right)}\right).$$

In the decoder, transposed convolutions are used for upsampling. The upsampled features are concatenated with the corresponding encoder features via skip connections:

(Equation 7) $$F_{\text{up}}^{\left(l\right)}=UpConv\left(F^{\left(l+1\right)}\right)⊕F_{\text{skip}}^{\left(l\right)}.$$

A final 1 × 1 convolution maps the feature maps to a three-channel prediction output $\hat{Y}$.

To enhance the network’s ability to model both local structures and global context, we integrate multi-head self-attention (MHSA) modules into each encoder layer after the convolutional operation. Given an input feature map $F^{\left(l\right)}\in R^{C\times H\times W}$, we first reshape it into a sequence $X\in R^{C\times N}$, where *N* = *H* × *W*. This representation serves as the input to the attention mechanism. For each head *h* ∈ {1, *…*, *H*}, we project the input into query, key, and value matrices:

(Equation 8) $$Q^{\left(h\right)}=W_{Q}^{\left(h\right)}X,\;K^{\left(h\right)}=W_{K}^{\left(h\right)}X,\;V^{\left(h\right)}=W_{V}^{\left(h\right)}X.$$

The attention weights for the *h*-th head are calculated as:

(Equation 9) $$A^{\left(h\right)}=Softmax\left(\frac{{\left(Q^{\left(h\right)}\right)}^{⊤}K^{\left(h\right)}}{\sqrt{d_{k}}}\right).$$

The attended features for each head are then:

(Equation 10) $$Y^{\left(h\right)}=A^{\left(h\right)}V^{\left(h\right)}.$$

The outputs from all *H* attention heads are concatenated and linearly projected:

(Equation 11) $$Y=W_{O}\left[Y^{\left(1\right)};Y^{\left(2\right)};\ldots ;Y^{\left(H\right)}\right].$$

A residual connection is added to preserve original spatial features:

(Equation 12) $$F_{\text{attn}}^{\left(l\right)}=Y+X.$$

Finally, the reshaped $F_{\text{attn}}^{\left(l\right)}$ is fed into the max-pooling layer. The self-attention-enhanced feature map thus replaces the original **F**^(*l*)^ for downstream processing.

The proposed network incorporates several architectural innovations that enhance deformation representation learning. Progressive Channel Expansion: The encoder gradually expands feature channels from 3 to 1024, enabling hierarchical modeling of multi-scale anatomical structures and complex soft-tissue deformations. Hierarchical Attention Fusion: Multi-head self-attention, coupled with skip connections, captures both local contour boundaries and global contextual information, improving robustness under occlusion, illumination shifts, and imaging noise. Symmetric Residual Integration: Dense residual shortcuts merge fine-grained structural cues with semantic features, supporting precise contour and texture reconstruction. Lightweight Pyramid Decoder: A gradually narrowing decoder path (1024 → 64) allows efficient feature decoding with reduced redundancy, facilitating near real-time performance. Collectively, these design elements overcome the computational inefficiency and limited adaptability of traditional contour-based approaches. The framework minimizes reliance on extensive manual labels while improving interpretability, allowing automatic and accurate extraction of deformation regions and high-level semantic features for subsequent clinical analysis. Our model employs an end-to-end, multi-task generative paradigm that learns continuous semantic embeddings rather than discrete segmentation masks. Instead of binary classification, it reconstructs smooth-valued outputs—such as contours, heatmaps, and texture fields—directly from learned latent codes. These latent variables function similarly to deformation fields in classical B-spline registration but are optimized implicitly through the expressive capacity of deep encoder–decoder networks rather than hand-engineered basis functions. To enforce precise pixel-level correspondence, a mean squared error (MSE) loss is applied between the predicted map $\hat{M}\in {\left[0,1\right]}^{H\times W\times 1}$ and the ground truth $I\in R^{H\times W\times 1}$ across all channels:

(Equation 13) $$L_{\text{MSE}}=\frac{1}{N}\overset{N}{\underset{i=1}{\sum }}\frac{1}{C\cdot H\cdot W}\overset{C}{\underset{c=1}{\sum }}\overset{H}{\underset{h=1}{\sum }}\overset{W}{\underset{w=1}{\sum }}{\left({\hat{y}}_{i}\left(c,h,w\right)-y_{i}\left(c,h,w\right)\right)}^{2}.$$

where *N* denotes the batch size, and *C*, *H*, and *W* are the number of channels, height, and width, respectively. ${\hat{y}}_{i}$ and *y*_*i*_ represent the predicted and reference images for the *i*-th sample. This objective encourages accurate reconstruction by minimizing the Euclidean distance between prediction and reference. The formulation treats image generation as a dense continuous mapping, where minimizing MSE corresponds to maximizing the likelihood under an independent Gaussian noise assumption.

(Equation 14) $$L_{\text{Gaussian}}=-\log p\left({\hat{y}}_{i}|y_{i}\right)=\frac{1}{2\sigma^{2}}{\left\|{\hat{y}}_{i}-y_{i}\right\|}^{2}+\text{const}.$$

In the proposed encoder–decoder architecture, deformation contours are implicitly captured through neuron activations. The encoder hierarchically compresses geometric features into a latent space, enabling semantic abstraction of input shapes, while the decoder generatively reconstructs structural details from the high-level embedding. This unsupervised framework eliminates manual annotations and embeds contour information into a compact latent representation via fully connected layers, enabling efficient and robust modeling of deformation semantics analogous to learning a B-spline like field through neural approximation.

#### Deformation detection based tracking via multi-feature fusion

We propose a hierarchical, multimodal tracking framework guided by explicit masks to robustly localize soft tissue deformations under challenging conditions such as blurred edges, low-texture regions, and non-rigid motion. The framework generates augmented template regions through affine and nonlinear transformations, producing multiple candidate templates with three complementary modalities: **contour** (structural cues), **color** (appearance), and **heatmap** (semantic information). Before matching, each candidate template is multiplied element-wise with its corresponding mask to retain only the target region, improving structural consistency and robustness against deformation. Let *I*_*t*_ denote a candidate region from the current frame. Similarity is computed independently for the short-term template *T*_*s*_ and the long-term template *T*_*l*_:

(Equation 15) $$S_{s}=\text{Sim}\left(I_{t},T_{s}\right),S_{l}=\text{Sim}\left(I_{t},T_{l}\right)$$

The multimodal similarity function Sim(·) fuses contour, color, and heatmap features:

(Equation 16) $$\text{Sim}\left(A,B\right)=\lambda_{1}\;\text{NCC}\left(A_{\text{color}},B_{\text{color}}\right)+\lambda_{2}\;\text{Dice}\left(A_{\text{edge}},B_{\text{edge}}\right)+\lambda_{3}\;\text{HMScore}\left(A_{\text{heat}},B_{\text{heat}}\right)$$

where NCC measures color correlation, Dice evaluates contour overlap, and HMScore measures heatmap similarity. The weights *λ*_1_, *λ*_2_, *λ*_3_ control the contribution of each feature. The final fused similarity score is obtained via a weighted combination of short- and long-term responses:

(Equation 17) $$S_{f}=\alpha S_{s}+\left(1-\alpha \right)S_{l}$$

where *α* ∈ [0, 1] is a dynamic memory fusion weight, updated according to tracking confidence or temporal distance from initialization.

To quantify geometric deformations between consecutive frames, we introduce a contour-based Delaunay triangulation method. First, contour landmarks are extracted from the target region to form a set of points: P. A Delaunay triangulation is then performed over these points to generate triangles: T.

(Equation 18) $$P=\left\{p_{i}\in R^{2}\right\},T=\text{Delaunay}\left(P\right).$$

Each triangle ${△}_{i,j,k}\in T$ corresponds to a local region whose deformation is approximated by an affine transformation matrix: $A_{i,j,k}\in R^{2\times 2}$. The affine matrix *A*_*i*,*j*,*k*_ is determined by minimizing the squared distance between the corresponding triangle vertices across consecutive frames:

(Equation 19) $$A_{i,j,k}=\arg\min_{A}\underset{v\in \left\{i,j,k\right\}}{\sum }\|Ap_{v}-p_{v}^{\prime }{\|}^{2}.$$

where *p*_*v*_ and $p_{v}^{\prime }$ represent the vertex coordinates in the previous and current frames, respectively. This approach enables precise modeling of local deformations and supports the construction of dense deformation fields for detailed morphometric analysis.

To robustly track soft tissue deformations, we propose a hierarchical, multimodal feature fusion framework guided by explicit masking. Given a video stream ${\left\{I_{t}\right\}}_{t=0}^{T}$ and an initial ROI *R*_0_, the framework generates a dynamic template library T and outputs the target trajectory ${\left\{\left(x_{t},y_{t}\right)\right\}}_{t=1}^{T}$. A set of *L* candidate templates is generated from the initial ROI via affine and nonlinear transformations. For each template:

(Equation 20) $$\begin{array}{cc} \theta & ∼U\left(-30{}^{\circ},30{}^{\circ}\right),\;s_{x},s_{y}∼U\left(0.35,1.5\right),\\ \Delta A & ∼N\left(0,0.1I_{2}\right),\;A_{i}=R\left(\theta \right)S\left(s_{x},s_{y}\right)+\Delta A,\\ T_{i} & =W\left(R_{0};A_{i}\right),\;\left(\phi_{i}^{c},\phi_{i}^{e},\phi_{i}^{h}\right)=f_{\theta }\left(T_{i}\right). \end{array}$$

where *R*(*θ*), *S*(*s*_*x*_, *s*_*y*_), and Δ*A* denote rotation, scaling, and affine perturbation matrices, respectively; W is the warping operator; and *f*_*θ*_(·) extracts contour, color, and heatmap features. Each template is stored in T.

During matching, we leverage spatiotemporal continuity and locality. Empirical analysis of semi-HD videos shows that object displacement typically ranges from 16 to 64 pixels. Accordingly, a sliding-window multi-channel search is conducted around the target bounding box in the current frame. Temporal consistency is ensured via short and long-term memory models. Template localization is refined using normalized cross-correlation, and deformation is analyzed through Delaunay triangulation of extracted contours. For each frame *t*, a search region *S*_*t*_ is constructed around the previous ROI:

(Equation 21) $$S_{t}=\text{Pad}\left(R_{t-1},32\right),$$

and features are extracted:

(Equation 22) $$\left(\Phi_{t}^{c},\Phi_{t}^{e},\Phi_{t}^{h}\right)=f_{\theta }\left(S_{t}\right).$$

Modality specific metrics are adopted: Euclidean distance for color, Dice coefficient for contour overlap, and weighted MSE for heatmap differences. A confidence weighted fusion strategy adaptively integrates these metrics, followed by threshold based candidate selection. Similarity between each candidate template $\left(\phi_{i}^{c},\phi_{i}^{e},\phi_{i}^{h}\right)\in T$ and the current frame is computed via:

(Equation 23) $$s_{i}=\underset{k}{\sum }\omega_{k}\cdot \text{sim}\left(\phi_{i}^{k},\Phi_{t}^{k}\right),$$

where sim(·) represents modality-specific metrics: Euclidean distance for color, Dice coefficient for contour overlap, and weighted MSE for heatmaps. The best candidate is selected as:

(Equation 24) $$i^{*}=\arg\max_{i}s_{i},$$

and the ROI is updated if $s_{i^{*}}$ exceeds a threshold. The template library is dynamically updated while maintaining a fixed size. Multimodal feature fusion is performed by concatenating contour, color, and heatmap embeddings:

(Equation 25) $$F_{\text{fusion}}=\text{Concat}\left[\psi \left(F_{\text{contour}}\right),\psi \left(F_{\text{color}}\right),\psi \left(F_{\text{heatmap}}\right)\right]\in R^{64\times 64\times 9}.$$

The search window is dynamically adjusted:

(Equation 26) $$W_{t}=\left\{\begin{array}{c} W_{\text{base}},\text{if}\;\text{motion}\;\text{coherence}\;\text{is}\;\text{satisfied},\\ W_{\text{base}}⊕\text{disk}\left(r_{t}\right),\;r_{t}=\left\lfloor 2\sigma_{t}\right\rfloor ,\text{otherwise}. \end{array}\right)$$

#### Deep metric learning network for contour shape similarity

Let $I^{\left(t\right)}\in R^{H\times W}$ denote the input frame at time *t*, and $M^{\left(t\right)}\in {\left\{0,1\right\}}^{H\times W}$ be the corresponding binary segmentation mask. The contour $C^{\left(t\right)}=\partial M^{\left(t\right)}$ is defined as the boundary of the support region $M^{\left(t\right)}$. Given the template contour $C_{\text{tmpl}}$ from the initial frame *t* = 1, the tracking problem is formulated as finding the optimal transformation Φ^(*t*)^ that maximises the similarity between $C_{\text{tmpl}}$ and the candidate contour at frame *t*:

(Equation 27) $$\Phi^{\left(t\right)*}=\arg\max_{\Phi \in T}\;S_{\theta }\left(C_{\text{tmpl}},\;\Phi \left(C^{\left(t\right)}\right)\right),$$

where T is the search space of spatial transformations (translation and scale), and $S_{\theta }:C\times C\to \left[0,1\right]$ is a learned similarity function parameterised by *θ*, implemented via the ContourSimilarityNet.

The contour-based formulation provides three key advantages for surgical target tracking. (1) Illumination invariance: The binary contour representation $C={\left\{\left(x_{i},y_{i}\right)\right\}}_{i=1}^{N}$ discards photometric information, making the tracker invariant to intensity transformations $I^{\prime }=\alpha I+\beta$. (2) Occlusion robustness: For partial visibility ratio $\rho =|C\cap C_{\text{gt}}|/|C_{\text{gt}}|$, the training objective enforces smooth similarity degradation $S_{\theta }\left(C,C_{\text{gt}}\right)\approx 0.08+0.92\cdot \rho^{0.85}$, providing graceful performance decay under occlusion. (3) Computational efficiency: The lightweight network architecture (1.2 × 10^6^ parameters) processes contour pairs in 2.3 ms on CPU, satisfying real-time constraints without GPU acceleration.

#### Temporal tracking algorithm

The tracking procedure is formulated as a sequential optimisation problem over frames *t* = 1, *…*, *T*. At *t* = 1, the template contour $C_{\text{tmpl}}$ is initialised from the ground-truth mask $M^{\left(1\right)}$ by boundary extraction $C_{\text{tmpl}}=\partial M^{\left(1\right)}$. To handle viewpoint variations, the template is augmented into a dictionary $D_{\text{tmpl}}={\left\{\Phi_{j}\left(C_{\text{tmpl}}\right)\right\}}_{j=1}^{173}$ through geometric transformations Φ_*j*_ encompassing rotation (*α* ∈ [−28°, 28°]), translation (Δ ∈ [−12%, 12%]), and warp perturbation (*ω* ∈ [3%, 10%]).

For each subsequent frame *t* > 1, target localisation proceeds via hierarchical optimisation. The coarse search stage evaluates all 173 augmented templates at reduced resolution (*S*′ = 32) across a spatial grid $G^{\left(t\right)}=\left\{\left(x_{0}+4k,y_{0}+4l\right)|k,l\in Z\right\}$ within radius *R* = 50 px from the previous location (*x*_0_, *y*_0_):

(Equation 28) $$\left(x^{*},y^{*},j^{*}\right)=\arg\max_{\left(x,y\right)\in G^{\left(t\right)},\;j\in \left\{1,\ldots ,173\right\}}S_{\theta }\left(\Phi_{j}\left(C_{\text{tmpl}}\right),\;C^{\left(t\right)}\left(x,y\right)\right),$$

where $C^{\left(t\right)}\left(x,y\right)$ is the contour extracted from the candidate region centered at (*x*, *y*) via thresholding (*τ* = 68) and connected component analysis.

An adaptive early exit mechanism terminates the search when confidence exceeds *η*_exit_ = 0.95, accepting (*x*∗, *y*∗) as the final result. This criterion $S_{\theta }^{\text{coarse}}\geq \eta_{\text{exit}}$ is satisfied in 60% of frames under stable tracking conditions, yielding a 60% reduction in computational cost. For ambiguous cases $\left(S_{\theta }^{\text{coarse}}<\eta_{\text{exit}}\right)$, the algorithm refines the top-*K* = 3 coarse candidates through local optimisation at full resolution (*S* = 128) with unit stride:

(Equation 29) $$\left(x^{*},y^{*}\right)=\arg\max_{\left(x,y\right)\in N_{1}\left(x_{k},y_{k}\right)}S_{\theta }\left(\Phi_{j^{*}}\left(C_{\text{tmpl}}\right),\;C^{\left(t\right)}\left(x,y\right)\right),$$

where $N_{1}\left(x_{k},y_{k}\right)=\left\{\left(x,y\right):|x-x_{k}|\leq 2,\;|y-y_{k}|\leq 2\right\}$ defines a 5 × 5 neighborhood around each candidate.

To accommodate gradual shape variations while maintaining temporal consistency, the template undergoes adaptive updates via exponential moving average when tracking confidence $S_{\theta }^{\left(t\right)}\geq \eta_{\text{update}}=0.90$:

(Equation 30) $$C_{\text{tmpl}}^{\left(t+1\right)}=\left(1-\lambda \right)\cdot C_{\text{tmpl}}^{\left(t\right)}+\lambda \cdot C^{\left(t\right)},$$

with update rate *λ* = 0.05. This mechanism ensures the template evolves with the target’s shape trajectory while preventing model drift during occlusion events $\left(S_{\theta }^{\left(t\right)}<\eta_{\text{update}}\right)$, where updates are suspended.

##### Dataset construction rationale

Existing endoscopic tracking datasets, including TrackVes, CholecSeg8k, SurgT-benchmarking, and EndoVis Instrument Tracking, rely on manual or semi-automatic annotation, which introduces subjectivity and potential inaccuracies in ground-truth labels. Boundary definitions for deformable surgical target under specular reflections, smoke, and fluid occlusion remain ambiguous, making quantitative comparisons between methods difficult to interpret. To address these limitations, we construct a standardised, controllable evaluation dataset using chessboard markers with three different specifications: 11 × 8 squares (11 mm per square), 9 × 8 squares (8 mm), and 9 × 6 squares (5 mm). The chessboard serves as a ground-truth reference with several advantages:

Pixel-accurate ground truth: Corner detection algorithms localise chessboard vertices with sub-pixel precision, enabling automatic generation of bounding boxes and binary masks without manual annotation. The geometric constraints of the chessboard pattern provide inherent validation of annotation correctness. Controlled challenge variations: By capturing sequences under different conditions, we systematically introduce tracking challenges including rotation (target articulation), translation (camera motion), scale changes (zoom, depth variation), partial occlusion (object collisions), and illumination changes (specular highlights, shadow casting). Reproducibility: The dataset construction pipeline is fully automated, from image capture through chessboard detection to ground-truth export, ensuring consistency across all 11 sequences (3,848 frames total) and enabling other researchers to replicate the evaluation protocol.

This controlled evaluation framework allows us to assess tracking methods under known ground-truth conditions, providing objective measurements of accuracy, precision, and robustness across varying challenge profiles.

##### Contour similarity network architecture

The proposed contour-based tracker employs a lightweight Siamese network, to measure shape correspondence between template and search region contours. The network consists of a shared encoder that maps binary contour images to L2-normalised embedding vectors, followed by cosine similarity computation. The encoder architecture implements a hierarchical feature extraction pipeline through *L* = 4 convolutional blocks with batch normalisation and ReLU activation. Given an input contour image **X**^(0)^ = **I** ∈ [0,1]^*S*×*S*^ with *S* = 128, the *ℓ*-th layer computes:

(Equation 31) $$X^{\left(\ell \right)}=\sigma \left({\text{BN}}_{\ell }\left(\overset{C_{\ell -1}}{\underset{c^{\prime }=1}{\sum }}W^{\left(\ell ,c^{\prime }\right)}*X^{\left(\ell -1,c^{\prime }\right)}+b^{\left(\ell \right)}\right)\right),\;\ell =1,\ldots ,L,$$

where $X^{\left(\ell \right)}\in R^{H_{\ell }\times W_{\ell }\times C_{\ell }}$ denotes the feature map at layer *ℓ* with *C*_*ℓ*_ channels, ∗ represents the 2D convolution operator, ${\text{BN}}_{\ell }\left(x\right)=\gamma_{\ell }\frac{x-\mu_{\ell }}{\sqrt{\sigma_{\ell }^{2}+\varepsilon^{\prime }}}+\beta_{\ell }$ is the batch normalisation operation with learnable parameters *γ*_*ℓ*_, *β*_*ℓ*_, *σ*(*x*) = max(0, *x*) is the ReLU activation function, and $W^{\left(\ell \right)}\in R^{C_{\ell }\times C_{\ell -1}\times K_{\ell }\times K_{\ell }}$ are the convolutional filter weights with kernel size *K*_*ℓ*_.

The network architecture follows a spatial pyramid structure with progressive dimensionality reduction and channel expansion:

(Equation 32) $$\begin{array}{cc} \text{Layer}\;1\text{:} & \;W^{\left(1\right)}\in R^{32\times 1\times 5\times 5},\;s=2\;\Rightarrow \;X^{\left(1\right)}\in R^{64\times 64\times 32},\\ \text{Layer}\;2\text{:} & \;W^{\left(2\right)}\in R^{64\times 32\times 3\times 3},\;s=2\;\Rightarrow \;X^{\left(2\right)}\in R^{32\times 32\times 64},\\ \text{Layer}\;3\text{:} & \;W^{\left(3\right)}\in R^{\mathrm{128\times 64\times 3\times 3}},\;s=2\;\Rightarrow \;X^{\left(3\right)}\in R^{16\times 16\times 128},\\ \text{Layer}\;4\text{:} & \;W^{\left(4\right)}\in R^{\mathrm{128\times 128\times 3\times 3}},\;s=2\;\Rightarrow \;X^{\left(4\right)}\in R^{8\times 8\times 128}. \end{array}$$

This hierarchical design achieves a spatial compression ratio of 2^4^ = 16 (from 128 × 128 to 8 × 8) while expanding the channel dimension from 1 to 128, enabling the network to capture multi-scale shape features from fine-grained boundary details to global contour context. The final feature tensor $X^{\left(4\right)}\in R^{8\times 8\times 128}$ is vectorised via row-major flattening:

(Equation 33) $$f=\text{Vec}\left(X^{\left(4\right)}\right)\in R^{d_{\text{flat}}},\;d_{\text{flat}}=8\times 8\times 128=8192,$$

and projected into the latent embedding space through a fully-connected layer:

(Equation 34) $$z=\frac{W_{\text{fc}}f+b_{\text{fc}}}{{\left\|W_{\text{fc}}f+b_{\text{fc}}\right\|}_{2}+\varepsilon },$$

where $W_{\text{fc}}\in R^{d\times d_{\text{flat}}}$ with *d* = 128, $b_{\text{fc}}\in R^{d}$, and *ϵ* = 10^−6^ ensures numerical stability. The *ℓ*_2_-normalisation operator $N\left(v\right)=v/\left(\|v{\|}_{2}+\varepsilon \right)$ constrains the embedding to lie on the unit hypersphere $z\in S^{d-1}\subset R^{d}$, satisfying ‖**z**‖_2_ = 1 and enabling cosine similarity computation in the embedding space.

#### Similarity computation

The Siamese architecture processes two input contours **I**_1_ and **I**_2_ through the shared encoder to obtain embeddings **z**_1_ and **z**_2_. The cosine similarity is computed as:

(Equation 35) $$\text{cos}\left(z_{1},z_{2}\right)=z_{1}^{⊤}z_{2}=\overset{128}{\underset{i=1}{\sum }}z_{1,i}\cdot z_{2,i},$$

which ranges from −1 to 1 due to L2 normalisation. The final similarity score is linearly mapped to [0, 1]:

(Equation 36) $$\text{sim}\left(I_{1},I_{2}\right)=\frac{\text{cos}\left(z_{1},z_{2}\right)+1}{2}.$$

A score of 1.0 indicates perfect contour match, while 0.0 indicates maximum dissimilarity.

#### Training strategy

The network is trained using mean squared error (MSE) loss between predicted similarity and target similarity:

(Equation 37) $$L_{\text{MSE}}=\frac{1}{N}\overset{N}{\underset{i=1}{\sum }}{\left(\text{sim}\left(I_{1}^{\left(i\right)},I_{2}^{\left(i\right)}\right)-y^{\left(i\right)}\right)}^{2},$$

where *N* is the batch size and *y*^(*i*)^ ∈ [0, 1] is the target similarity.

Positive pairs are generated from the same ground-truth mask contour with random augmentations: rotation (±28°), scale (0.82–1.18), translation (±12%), perspective perturbation (3–10%), and elastic deformation (*σ* = 2.5–5.0, *α* = 6.0–14.0). With occlusion probability 0.42, random rectangular masks are applied, and the target similarity is computed as:

(Equation 38) $$y=0.08+0.92\cdot {\left(\text{visible\_ratio}\right)}^{0.85},$$

where visible_ratio ∈ [0, 1] is the fraction of unoccluded foreground pixels. Without occlusion, y∼U(0.94,1.0).

Negative pairs are sampled from different sequences or frames, with target similarity y∼U(0.04,0.16). The training dataset contains 24, 576 samples per epoch with positive pair probability 0.58, trained for 180 epochs using AdamW optimizer (lr = 3 × 10^−4^, weight_decay = 10^−4^) with cosine annealing scheduler.

##### Augmentation strategy for robustness

To enhance generalisation under challenging surgical environments, the training procedure incorporates a comprehensive augmentation pipeline that systematically introduces geometric, topological, and photometric variations. Geometric transformations encompass affine operations (rotation ±28°, scale 0.82–1.18, translation ±12%) and perspective warping (3–10% boundary perturbation), simulating viewpoint changes and target articulation during endoscopic procedures. Non-rigid deformations are modeled through elastic warping with Gaussian smoothing (*σ* ∈ [2.5, 5.0]) and displacement fields (*α* ∈ [6.0, 14.0]), capturing contour shape variations arising from target flexure. Partial occlusion is synthesised via random rectangular masks covering 12–55% of the target area, with the training target computed as *y* = 0.08 + 0.92 · *ρ*^0.85^ where *ρ* is the visible foreground ratio, enforcing smooth similarity degradation proportional to visibility. Photometric variations are introduced through intensity perturbation of the grayscale input, simulating the illumination fluctuations and specular reflections characteristic of endoscopic imaging. This multi-faceted augmentation strategy generates 24, 576 diverse training samples per epoch, with positive pairs sampled at probability 0.58 and negative pairs at 0.42, ensuring the network learns discriminative shape representations robust to the full spectrum of intra-operative challenges. This controlled evaluation framework allows us to assess tracking methods under known ground-truth conditions, providing objective measurements of accuracy, precision, and robustness across varying challenge profiles.

In summary, the proposed contour-based tracking framework integrates hierarchical template matching with learned shape similarity metrics for robust deformable object tracking. The method operates through a unified optimisation pipeline that combines contour shape representation, multi-scale search, and adaptive template maintenance. The Siamese similarity network maps contour pairs to scores in [0, 1] with occlusion-aware training, enabling illumination-invariant tracking under partial visibility. The hierarchical coarse-to-fine search reduces computational cost by 60% through adaptive early exit, while mask-guided candidate generation ensures spatially coherent proposals. Template evolution via exponential moving average captures long-term deformation patterns, and the extracted contours support downstream deformation analysis through Delaunay triangulation for interpretable geometric characterisation. Through the integration of these components, the framework provides an efficient solution for surgical target tracking, while maintaining tracking accuracy across challenging occlusion and illumination scenarios.

### Quantification and statistical analysis

Data processing and analysis in this study were performed using CUDA and Python. For the boundary/edge and mask-segmentation benchmarks (Tables 1, 2, 4, and 5), each dataset-level score is the arithmetic mean of the corresponding per-image metric over the evaluated set, and we report it as mean_±SD_, where the standard deviation is taken over the per-image values and *n* is the number of evaluated images per split (BIPEDv2 *n* = 200/50 train/test; BSDS500 *n* = 200/100/200; SBD *n* = 8, 498/2, 857; CholecSeg8k *n* ≤ 8, 080; Kvasir-Instrument *n* ≤ 590; for methods with missing predictions *n* is smaller and is noted in the corresponding caption). Statistical significance is assessed with the two-sided Wilcoxon signed-rank test computed on the paired per-image values of our method and each baseline over their commonly evaluated images (∗*p* < 0.05, ∗∗*p* < 0.01), and is annotated by superscripts in the tables. For the chessboard tracking comparison (Table 6), the coverage-aware BBox IoU is aggregated per sequence and reported as mean_±SD_ over the 11 sequences, and statistical significance of our method versus each baseline is assessed with the paired two-sided Wilcoxon signed-rank test on the per-sequence IoU. For the in-house deformation-region segmentation (Table 3), scores are reported as per-frame means over the evaluated frames; per-sample dispersion for every compared baseline was not uniformly retained in this setting, so we report the means and note this as a reporting limitation.
